# Silica nanoparticles alleviate cadmium toxicity to *Pisum sativum* L. seedling growth by remodeling carbon-nitrogen metabolism

**DOI:** 10.3389/fpls.2025.1641785

**Published:** 2025-10-23

**Authors:** Xiaohuan Yang, Weifeng Zhao, Hongxin Li, Lingling Sun, Liyin Wang, Ziran Wang, Jingyi Yang, Baoqiong Zhang, Liangyi Zhao, Xibin Zhang, Liangliang Sun, Jinhu Ma

**Affiliations:** ^1^ College of Agricultural, Shanxi Agricultural University, Taigu, China; ^2^ College of Tropical Crop, Yunnan Agricultural University, Kunming, China; ^3^ Agricultural and Rural Bureau of Fenyang, Lvliang, China; ^4^ Shenyang Institute of Technology, Fushun, China; ^5^ State Key Laboratory of Crop Gene Exploration and Utilization in Southwest China, Sichuan Agricultural University, Chengdu, China; ^6^ College of Innovation and Entrepreneurship, Shanxi Agricultural University, Taigu, China

**Keywords:** cadmium toxicity, SiO2 nanoparticles, transcriptome analysis, sucrose metabolism, regulation pathway

## Abstract

The increasing incidence of soil cadmium (Cd) pollution significantly hinders the sustainable development of agriculture and food security. Improving crop stress resistance through nanobiotechnology represents a secure and sustainable approach for increasing the efficiency of treating soils contaminated with heavy metals. This study investigated the physiological and molecular mechanisms by which silica nanoparticles (nSiO_2_) alleviate plant Cd toxicity via ZW6 pea as the experimental material. These results indicate that Cd treatment severely impedes the growth and development of peas. However, nSiO_2_ application notably increased the lateral root number (25.00%), primary root length (33.93%), leaf dry weight (29.18%), root dry weight (17.41%), and photosynthesis rate (13.84%), thereby reducing the degree of oxidative damage caused by Cd toxicity. Moreover, Cd content in the roots (22.24%) and leaves (67.88%) of pea seedlings decreased with nSiO_2_ treatment, improving mineral nutrition and alleviating Cd-induced growth inhibition. Transcriptomic analysis revealed differentially expressed genes (DEGs) in pea seedlings subjected to Cd toxicity and nSiO_2_ treatment, revealing the molecular response of these plants to Cd stress. The addition of nSiO_2_ alongside Cd toxicity altered the C/N metabolic pathway in peas, particularly affecting sucrose and amino acid metabolism. This study highlights the effectiveness of nSiO_2_ in reducing Cd accumulation, mitigating oxidative stress, enhancing micronutrient absorption, restructuring metabolic pathways, and alleviating the growth inhibition caused by Cd toxicity. These findings provide a theoretical framework for enhancing crop stress resistance in agriculture through nanoparticle technology, offering a novel strategy for managing farmland contamination by heavy metals and promoting sustainable agricultural practices.

## 1 Introduction

The heavy metal cadmium (Cd) is widely recognized as one of the most toxic substances, occurring naturally in sulfide form within various metal mines (Waalkes and Diwan, 1999). Cd is a non-threshold toxin, and it can exert toxic effects even at extremely low concentrations (Rahman and Singh, 2019). The contamination of farmland soil with Cd resulting from industrial waste discharge has been increasing due to advancements in the heavy industry and mining sectors (Jayakumar et al., 2021). In recent years, the high concentrations of heavy metals such as Cd in the atmosphere, the lithosphere, the hydrosphere, and the biosphere have become a global problem, and they have severe destructive effects on various microorganisms, plants, and animals (Rahman and Singh, 2019). The radial movement of Cd element in plants is restricted by the plasmalemma barrier of roots and the chelation effects such as plant chelating peptides and vacuolar isolation (Asare et al., 2023). Once absorbed by plant roots, Cd accumulates in plants, hindering their normal growth and development, resulting in stunted plant stature, leaf chlorosis, and reduced root and stem length (Kumar and Aery, 2016). In crops, Cd competes with mineral elements for absorption and interferes with the physiological characteristics of plants, thereby inhibiting their growth in soil contaminated with Cd. The toxicity and carcinogenicity of Cd pose a serious threat to crop growth, productivity and human health (Aslam et al., 2023).

In contrast to the physical adsorption of metal ions (Li et al., 2025), the accumulation of Cd in plants occurs through root absorption, exerting a toxic effect on plant physiology. This toxicity disrupts the antioxidant system, leading to cellular damage in roots and stems (Kumar and Aery, 2016). Research has demonstrated that Cd stress significantly inhibits the growth characteristics of wheat, including the number of leaves per plant, the number of tillers per plant, biomass yield, stem/root length, and leaf area. It also significantly reduces the photosynthetic efficiency of wheat. Furthermore, Cd stress greatly reduces the contents of proline, ascorbic acid (AsA), glycine betaine (GB), tocopherol, total free amino acids (TFAA), and total soluble sugar (TSS) (Farhat et al., 2022). The concentration of Cd in the growth environment and the plant genotype determine the Cd accumulation characteristics of the plant. Transport proteins effectively mediate the process by which Cd is transported from the xylem and phloem to the above-ground part (Asare et al., 2023). Components such as hemicellulose, pectin, and polygalacturonic acid in the cell wall effectively bind with Cd^2+^, reducing their uptake by roots (Ma et al., 2015). Excessive Cd in the plant body will prompt the generation of secondary metabolites with antioxidant properties to counteract its toxic effects (Asare et al., 2023). At the molecular level, Cd triggers an increase in glutathione (GSH) levels in plants, acting as a precursor for the ASA-GSH cycle that aids in scavenging reactive oxygen species (ROS), maintaining homeostasis, and ensuring normal genetic processes under Cd-induced stress (Zechmann et al., 2008). Cd is sequestered either extracellularly or intracellularly in plant tissues after being transported from the roots to the aboveground parts. The ATP-binding cassette (ABC) transporter protein family, recognized as crucial for plant heavy metal detoxification, helps in handling Cd toxicity by sequestering chelated Cd.

Nanoparticles (NPs) exhibit surface and interface effects, as well as microsize effects, making them valuable tools in agricultural applications (Sajid and Płotka-Wasylka, 2020). Akpomie et al. (2023) elaborated on the adsorption mechanisms of heavy metal pollutants onto zinc oxide nanoparticles. They proposed that complexation, precipitation, ion exchange, and electrostatic interactions could serve as plausible mechanisms for adsorption onto zinc oxide nanoparticles, with complexation being identified as the dominant process. Silica nanoparticles (nSiO_2,_ Nano-SiO_2_) can promote plant growth and development while enhancing plant resilience to stress factors. Previous studies have explored how nSiO_2_ mitigate Cd stress in crops such as rice, barley, and maize. However, these studies have focused on monocotyledonous plants, primarily investigating how nSiO_2_ influences Cd tolerance through physiological response pathways (Ahmed et al., 2023; Anwar et al., 2025; Rhimi et al., 2024). Studies have shown that nSiO_2_ can significantly increase the concentration of photosynthetic pigments, improve cellular osmotic adjustment, and markedly enhance the activity of antioxidant enzymes such as SOD, CAT, and APX, thereby alleviating oxidative damage (Anwar et al., 2025). Nano-SiO_2_ can bind with chloroplasts in leaves, resulting in an increase in chlorophyll content, improved gas exchange, and increased photosynthesis in plants (Siddiqui et al., 2015). Research has indicated that nSiO_2_ effectively binds with Cd on the rice cell wall, thereby reducing Cd absorption under Cd stress conditions. Moreover, nSiO_2_ has been shown to suppress the expression of Cd transport-related genes such as *OsLCT1* and *OsNramp5* in rice, thus mitigating Cd-induced harm to plants (Cui et al., 2017). Nevertheless, the mechanism by which nSiO_2_ affects the response to Cd toxicity in legumes, particularly peas (*Pisum sativum* L.), remains unclear. Pea is selected as a model legume due to its economic importance as a global crop and its ecological relevance in sustainable agriculture. Although advanced adsorption modeling frameworks have been employed to quantify nanoparticle–metal interactions across various environmental contexts (Zeng et al., 2018), the application of such theoretical approaches remains markedly underexplored in leguminous plant systems such as peas—particularly under nSiO_2_ amendment—representing a critical knowledge gap in understanding the mechanistic basis of nanotechnology-enhanced phytoremediation. Consequently, our study delves into the physiological and molecular mechanisms behind nSiO_2_-mediated Cd accumulation in peas. In contrast, this study explores, through physiological mechanism analysis, how nSiO_2_ regulates the response of pea plants to Cd stress. Furthermore, by employing high-throughput sequencing technology, this research combines transcriptome analysis with physiological responses under nSiO_2_+Cd stress for the first time, to investigate the response patterns of peas to Cd stress. The specific objectives of this study are to: (1) evaluate the effects of nSiO_2_ on growth and physiological parameters under Cd stress; (2) analyze the transcriptomic changes in peas exposed to combined nSiO_2_ and Cd treatment; and (3) identify key pathways and genes involved in nSiO_2_-induced mitigation of Cd toxicity.

## 2 Materials and methods

### 2.1 Plant material and growth conditions

The pea (*Pisum sativum* L.) variety used in this study was Zhongwan 6 (ZW6). In this study, the Hoagland nutrient solution hydroponics method was employed for plant tissue culture and drug treatment. Pea seeds with complete grains and uniform sizes were carefully selected, followed by germination promotion at 28 °C in moist sand. Once the pea seedling roots reached a length of 0.5–1 cm (typically within 1–2 d), seedlings exhibiting similar growth patterns were chosen. The sand adhering to the root surface was subsequently rinsed with distilled water before being transferred into a 1/4-strength Hoagland nutrient solution (Xu et al., 2013). The hydroponic box was then placed on a light culture rack for seedling cultivation under specific conditions (light/dark: 18/6 h, temperature: 25/16 °C, humidity: 60%, light intensity: 20000 lux). During seedling cultivation, the nutrient mixture was replaced every 3 days. Ten-day-old pea seedlings were exposed to Cd (9 μM, cadmium chloride (CdCl_2_) was used as the source of Cd) or nSiO_2_ (50 mg/L) for 12 d. The selected concentrations of Cd and nSiO_2_ used in this experiment have been previously investigated and reported in existing studies (Głowacka et al., 2019; Ghosh et al., 2022). After 12 d of treatment, a range of physiological and biochemical analyses were conducted. Some samples were quickly frozen in liquid nitrogen after sampling and stored at -80 °C for later use.

### 2.2 The nSiO_2_ stock preparation

In this study, nSiO_2_ (purity 99%, size 50 ± 5 nm) was purchased from McLean Biotechnology Co., Ltd. Numerous studies have employed X-ray diffraction (XRD) to analyze the crystallinity of nSiO_2_, Fourier-transform infrared spectroscopy (FTIR) to detect its surface functional groups, and high-resolution transmission electron microscopy (HR-TEM) to characterize the shape, size, and morphology of nSiO_2_ in aqueous environments. XRD measurements were conducted on a Rigaku SmartLab diffractometer using Cu Kα radiation (λ = 1.5406 Å), with a scanning range from 10° to 80° (2θ), a step size of 0.02°, and a scanning speed of 2° per minute. The FTIR analysis was performed using a Thermo Scientific Nicolet iS10 spectrometer, with scans acquired over the range of 4000–400 cm⁻¹ at a resolution of 4 cm⁻¹, accumulating 32 scans per sample (Cui et al., 2017; Ghosh et al., 2022; Liu et al., 2025). nSiO_2_ was suspended in sterile deionized water (ddH_2_O), stirred for 2 h, and homogenized by ultrasonication at 40 kHz for 60 min until the NPs were evenly distributed, as described previously (Cui et al., 2017; Zou et al., 2022). The final working concentration of nSiO_2_ for plant treatment was 50 mg/L.

### 2.3 Phenotypic parameters

Plant growth parameters (including plant height, primary root (PR) length, leaf dry weight (DW), root DW, lateral root number, chlorophyll content and photosynthetic parameters) were measured after 12 d of Cd, nSiO_2_ or combined treatment. The roots were placed in a scanning dish, the pea root system was scanned via a scanner (EPSON Perfection V800 Photo), and the results were analyzed via WinRHIZO (Pro2016A). The chlorophyll content was measured via SPAD 502 (Minolta, Japan). At least three independent biological replicates were performed, with 25 plants measured in each treatment group.

### 2.4 Analysis of antioxidant enzyme activity

Total protein was extracted in potassium phosphate buffer (50 mM, pH 7.8) on ice. After centrifugation (15 min, 15000 rpm, 4 °C), the supernatant was removed for determination of SOD, CAT, and POD activities. SOD activity was measured as described by Gong et al. (2014) with a spectrophotometer. Briefly, samples were homogenized in ice-cold 50 mM phosphate buffer (pH 7.4). The assay system contained 50 mM phosphate buffer (pH 7.8), 100 μM EDTA, 50 mM xanthine, 24 μM NBT, and sample. The reaction was initiated by adding 50 mU/mL xanthine oxidase and incubated at 25 °C for 20 min. The absorbance of the formazan product was read at 560 nm using a spectrophotometer (Shimadzu UV-1800) (n = 3). One unit of SOD activity was defined as the amount of enzyme causing 50% inhibition of NBT reduction. Activity was normalized to protein concentration determined by BCA assay and expressed as U/mg protein. CAT activity was measured following Sun et al. (2020). The reaction mixture contained 50 mM phosphate buffer (pH 7.0), 30 mM H_2_O_2_, and enzyme extract. The decomposition of H_2_O_2_ was monitored by the decrease in absorbance at 240 nm for 1 min using a spectrophotometer (Shimadzu UV-1800) (n = 3). One unit of CAT activity was defined as the amount of enzyme that decomposed 1 μmol H_2_O_2_ per minute and was expressed as U/min/mg protein. POD activity was determined according to Sun et al. (2020). The assay contained 50 mM phosphate buffer (pH 7.0), 10 mM guaiacol, 5 mM H_2_O_2_, and enzyme extract. The increase in absorbance due to tetraguaiacol formation was recorded at 470 nm for 2 min using a spectrophotometer (Shimadzu UV-1800) (n = 3). One unit of POD activity was defined as the amount of enzyme that caused an increase of 0.01 in absorbance per minute and was expressed as U/min/mg protein.

### 2.5 Hydrogen peroxide determination

Hydrogen peroxide (H_2_O_2_) content was assayed as described by Gong et al. (2014) using a spectrophotometer. Briefly, samples were homogenized in cold acetone. The reaction mixture contained extract, 0.1% titanium sulfate, and 0.2 M H_2_SO_4_. After incubation at 25 °C for 10 min, the absorbance was measured at 415 nm using a spectrophotometer (Shimadzu UV-1800) (n = 3). H_2_O_2_ content was quantified against a standard curve and expressed as μmol/g fresh weight.

### 2.6 Root activity determination

In this study, the TTC method was employed to quantify root activity. Initially, 0.2 g of fresh pea root was accurately weighed and placed in a triangular bottle. A solution containing 0.5% TTC and 0.1 M phosphate buffer (pH=7.5) was subsequently added in equal volumes and thoroughly mixed with the sample before being incubated at 37 °C for 1 h. Following the incubation period, termination of the reaction was achieved by adding 1 M H_2_SO_4_ solution to the triangular bottle. The pretreated roots were then ground with ethyl acetate via a mortar, after which the absorbance value of the resulting liquid extract was measured at 485 nm following volume standardization (n = 3). Root activity was quantified on the basis of the reduction intensity of TTC.

### 2.7 Determination of photosynthetic characteristics

The leaves (No. 2–3 pairs) of pea plants subjected to different treatments were carefully selected, and the relevant photosynthetic data were measured via LI-6800 portable photosynthesis system (LI-COR) under natural light conditions. The intensity of photosynthetically active radiation (PAR) was set at 500 μmol·m^-2^·s^-1^, while the relative humidity (RH) was maintained at 50%. Measurements were taken for the net photosynthetic rate (Pn, μmol·m^-2^·s^-1^), stomatal conductance (Gsw, mol·m^-2^·s^-1^), and transpiration rate (Tr, mmol H_2_O·m^-2^·s^-1^). After 3 h in darkness to culture the pea plants, the chlorophyll fluorescence indices of the leaves were subsequently determined via pulse-modulated fluorometry equipment (Image-PAMM, Walz, Germany) with 25 plants measured in each treatment group.

### 2.8 Mineral element determination

After treatment, the roots and leaves of the pea plants in each group were collected. The samples were soaked in 1 mM EDTA solution for 30 min and then rinsed 5 times with ddH_2_O. The samples were subsequently fixed for 15 min at 105 °C and dried to a constant weight at 70 °C. The dried sample was ground and digested with HNO_3_ according to the methods of Sun et al. (2020). We initially measured a 0.2 g sample, which was subsequently transferred to a clean digestion tube for nitric acid (HNO_3_) digestion. The resulting solution was subsequently filtered through a microporous filter membrane (pore size 0.22 µm) before being transferred into a sample vial for measurement purposes. The contents of Cd, zinc (Zn), copper (Cu), magnesium (Mg), iron (Fe) and silicon (Si) were determined via inductively coupled plasma–mass spectrometry (ICP–MS). Each experiment was repeated three times.

### 2.9 Comparative transcriptome analysis

Ten-day-old pea seedlings were exposed to Cd, nSiO, or a combination of both. After a 2-day treatment and culture period, the roots and leaves of the peas in each treatment group were collected. Total RNA was extracted by grinding frozen samples with liquid nitrogen for subsequent transcriptomic high-throughput sequencing and qRT–PCR experiments (n = 3). The obtained transcriptome data were subjected to a bioinformatics analysis process provided by BMKCloud (www.biocloud.net). Differential expression analysis and functional annotation of differentially expressed genes (DEGs) via Kyoto Encyclopedia of Genes and Genomes (KEGG) were performed on the basis of gene expression levels in different sample groups. Gene reads represent gene expression levels, with fragments per kilobase of transcript per million fragments mapped (FPKM) values commonly used as a measure for convenience.

Genes exhibiting significantly different expression levels across samples are referred to as DEGs. During the process of detecting differentially expressed genes, the screening criteria were set as a fold change ≥ 2 and the FDR < 0.01. The fold change represents the ratio of expression between two samples, whereas the false discovery rate (FDR) is derived from correcting the significance p value (p-value), indicating the level of significance for differences observed. To facilitate comparison, the fold changes were paired and represented as log_2_FC values. The log_2_FC transformation was applied to normalize the gene expression data. The screening criteria for differentially expressed genes included a |log_2_(fold change)| ≥1 and q value ≤ 0.05.

### 2.10 Statistical analysis

Three independent biological replicates were used for each experiment in our study. The relevant figures were graphed with the software GraphPad Prism 8 and enhanced with Adobe Photoshop 2019 CC. The experimental results are shown as the means ± standard errors (SEs). The significance of differences was analyzed via Student’s *t* test (IBM SPSS Statistics 23.0). The asterisk indicates *P* < 0.05. One-way ANOVA with Tukey’s test was used to compare multiple groups. Different lowercase letters represent significant differences at *P* < 0.05.

## 3 Results

### 3.1 Effects of nSiO_2_ on the early growth of pea seedlings under Cd stress

**Figure 1:**
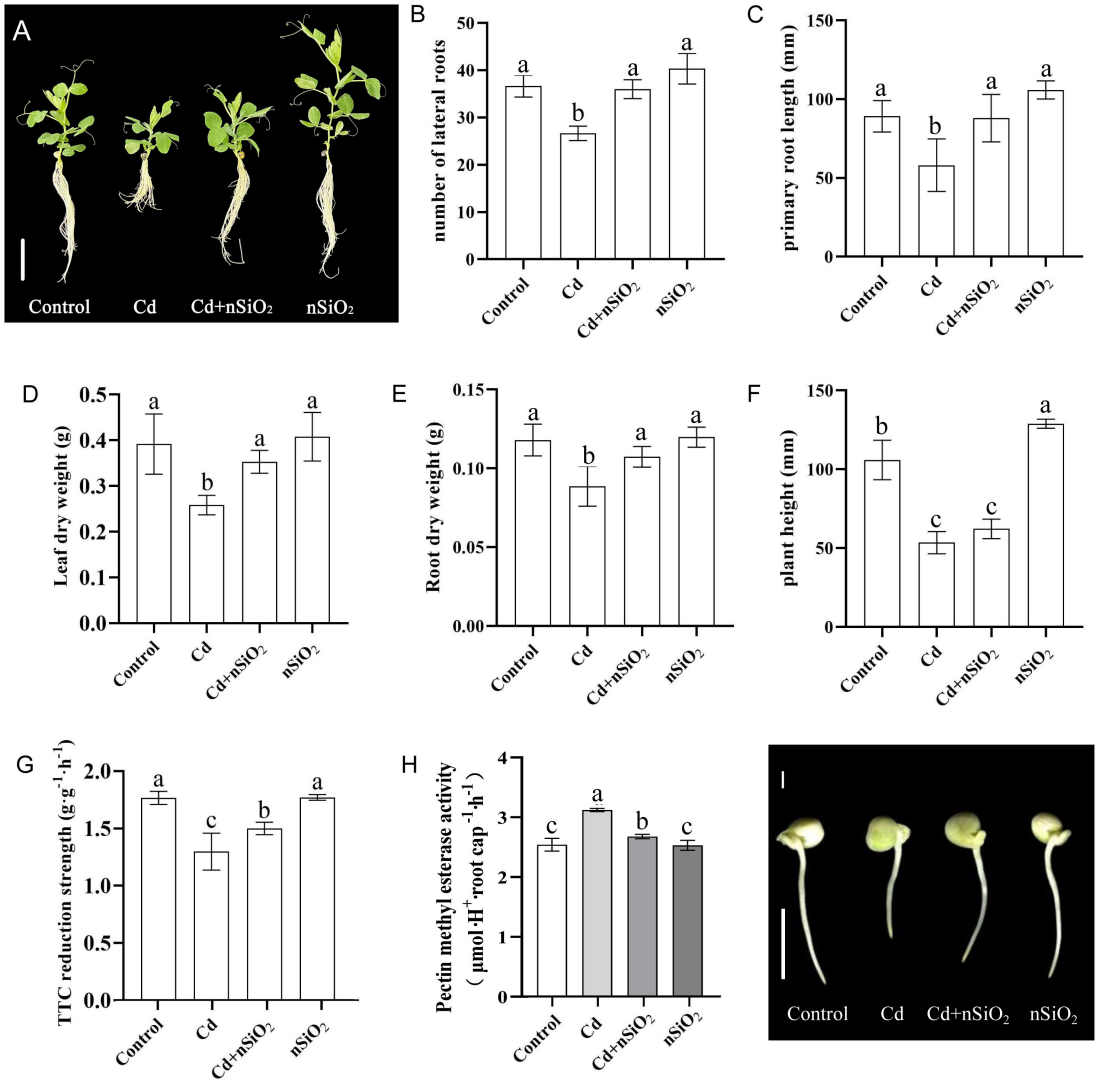
Nano-SiO_2_ alleviates Cd-mediated pea seedling growth inhibition. Ten-day-old pea plants were transferred to 1/4-strength fresh Hoagland solution with or without 9 μM CdCl_2_, 50 mg/L nSiO_2_ (Control, Cd, Cd+nSiO_2_, nSiO_2_)for 12 days (bar = 10 cm), **(A)** phenotypic images of pea seedlings under four treatments, **(B)** the lateral root number, **(C)** primary root length, **(D)** leaf dry weight (DW), **(E)** root DW, **(F)** plant height, **(G)** TTC (2,3,5-triphenyltetrazolium chloride) reduction strength, **(H)** Pectin Methyl esterase were measured. Two-day-old germinated pea seedlings were transferred to 1/4-strength fresh Hoagland solution with or without 9 μM CdCl_2_, 50 mg/L nSiO_2_ (Control, Cd, Cd+nSiO_2_, nSiO_2_) for a 24-hour treatment period. **(I)** The seed germination and radicle growth phenotype of peas (bar = 10 mm). The results shown are the means ± SDs (*n*=3), and different letters indicate significant differences (*P* < 0.05 according to Tukey’s test).

The effects of Cd on the growth of pea seedlings were analyzed in this study. The application of Cd adversely affected various growth parameters of the seedlings, resulting in reductions of 49.43% in plant height (compared with the control 105.89 ± 7.21 mm), 34.81% in primary root length (compared with the control 89.15 ± 5.77 mm), 27.03% in lateral root number (compared with the control 37 ± 1.33 roots), 39.87% in leaf dry weight (DW) (compared with the control 0.3958 ± 0.0230 g), and 24.83% in root DW (compared with the control 0.1180 ± 0.0058 g) (**Figures 1A–F**). In contrast, exposure to nSiO_2_ significantly increased the height of pea plants compared with those in the control group (**Figures 1A, F**). Furthermore, exposure to nSiO_2_ under Cd stress conditions stimulated the growth of pea seedlings. As shown in **Figures 1A–E**, the Cd+nSiO_2_ treatment group presented increases of 33.93% in primary root length, 25.00% in lateral root number, 29.18% in leaf DW, and 17.41% in root DW compared with those of the seedlings treated with Cd alone. Cd stress not only hindered leaf expansion in pea plants but also decreased the leaf count (**Figure 1A**). Notably, exposure to nSiO_2_ significantly increased the growth and development of pea leaves compared with those in the control group (**Figure 1A**). Exposure to nSiO_2_ under Cd stress conditions significantly promoted leaf growth (**Figures 1A, D**). Ultimately, exposure to nSiO_2_ increased Cd tolerance in pea seedlings, thereby increasing their overall growth and development.

On the other hand, Cd toxicity significantly inhibited the activity of pea seedling roots by 26.48% (**Figure 1G**). Compared with the control, exposure to nSiO_2_ had no significant effect on the root activity of pea seedlings (**Figure 1G**). When pea seedlings were exposed to nSiO_2_, the activity of pea seedling roots increased under Cd toxicity. As shown in **Figure 1G**, the group treated with ‘Cd+nSiO_2_’ presented a notable increase in root activity of 15.52% compared with that in the Cd-only treatment group. The activity of pectin methylesterase (PME) is crucial for protecting plant root tips from stress (Chen et al., 2018). Therefore, we investigated PME activity in pea root tips subjected to Cd toxicity, nSiO_2_ exposure, or combined Cd+nSiO_2_ treatment. Cd toxicity increased PME activity in pea seedling root tips by 22.85% (**Figure 1H**). Compared with the control, exposure to nSiO_2_ did not significantly affect PME activity in pea seedling root tips (**Figure 1H**). In contrast, exposure to nSiO_2_ significantly reduced PME activity in pea seedling root tips under Cd stress conditions. As depicted in **Figure 1H**, the ‘Cd+nSiO_2_’ group presented a substantial 14.23% decrease in PME activity in pea seedling root tips compared with the Cd treatment alone group. Moreover, Cd toxicity strongly inhibited both seed germination and radicle growth in peas; however, exposure to nSiO_2_ increased pea seed germination and radicle growth under Cd stress conditions (**Figure 1I**).

### 3.2 Effect of nSiO_2_ on the ROS content of pea seedlings under Cd stress

**Figure 2:**
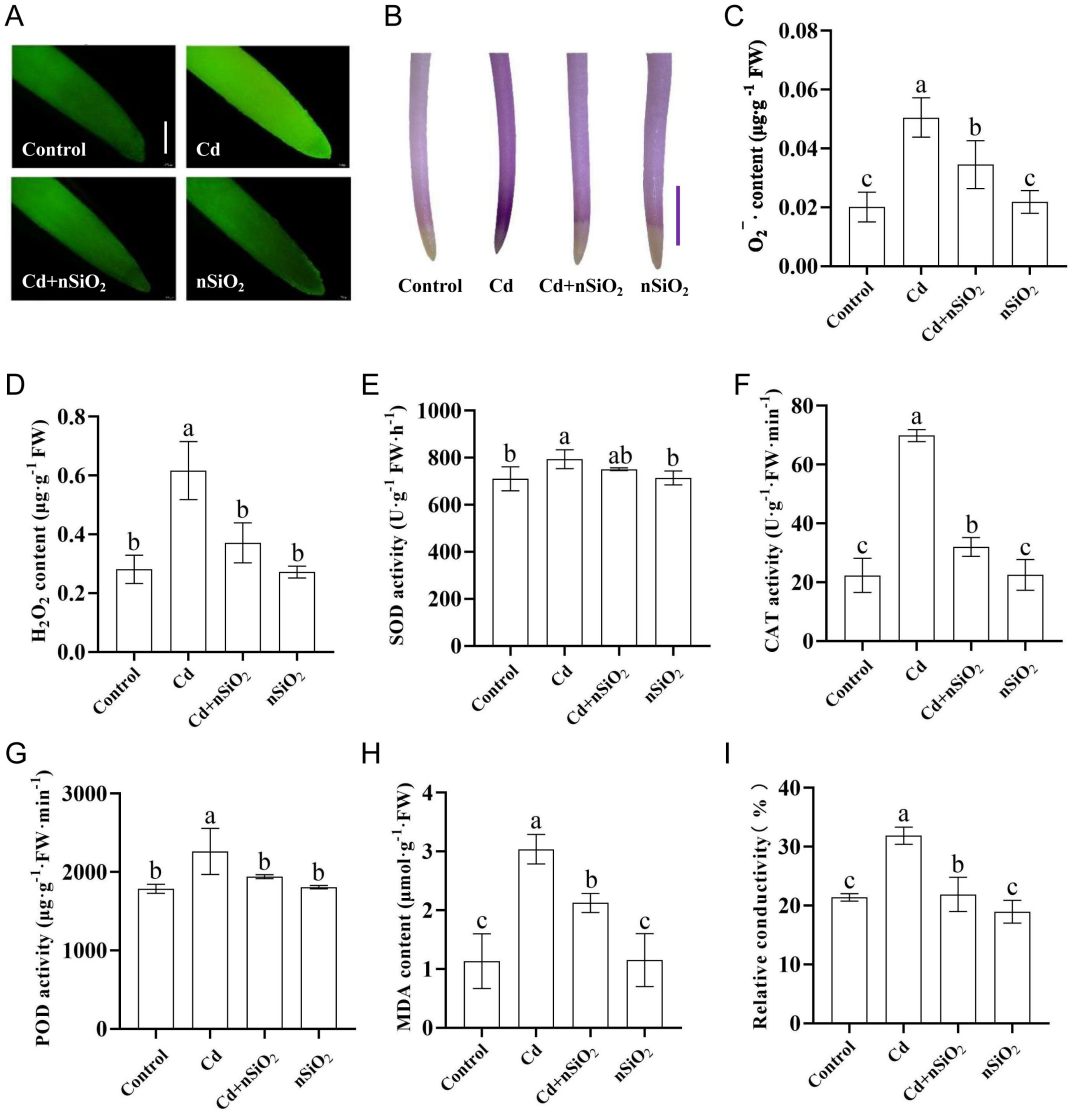
Nano-SiO_2_ exposure alleviated Cd-induced oxidative damage in pea seedling roots. Two-day-old germinated pea seedlings were transferred to 1/4-strength fresh Hoagland solution with or without 9 μM CdCl_2_, 50 mg/L nSiO_2_ (Control, Cd, Cd+nSiO_2_, nSiO_2_) for 5 days (bar = 100 μm). **(A)** Detection of ROS production in the roots of four treatments pea seedlings using the ROS-specific fluorescent probe DCFH-DA (bar = 100 μm) and **(B)** detection of O_2_^-^ production using NBT in pea roots (bar = 10 mm). The **(C)** O_2_¯ levels, **(D)** H_2_O_2_ levels, **(E)** SOD activity, **(F)** CAT activity, **(G)** POD activity, **(H)** MDA levels and **(I)** Relative electrical conductivity were measured. The results are presented as the means ± SDs (*n*=3). Different letters indicate significant differences (*P* < 0.05 according to Tukey’s test).

The accumulation of ROS serves as a critical indicator of plant responses to both biotic and abiotic stresses. In this study, we examined ROS accumulation in pea seedlings exposed to Cd toxicity, nSiO_2_ exposure, or a combination of both. Fluorescence staining for total ROS indicated a significant increase in ROS levels in the roots of pea seedlings under Cd toxicity (**Figure 2A**). NBT staining analysis of pea seedling roots revealed elevated superoxide anion (O_2_¯) levels under Cd toxicity (**Figure 2B**). Exposure to nSiO_2_ resulted in a reduction in ROS accumulation in pea roots under Cd stress (**Figures 2A, B**). Quantitative analysis revealed that under Cd stress, O_2_¯ and hydrogen peroxide (H_2_O_2_) levels in pea seedlings increased by 151.24%, 119.57%, 150.51%, and 73.96% in roots and leaves, respectively (**Figures 2C, D**; **Supplementary Figures S1A, B**). However, exposure to nSiO_2_ did not affect the levels of O_2_¯ or H_2_O_2_ in either the roots or leaves of pea seedlings compared with those in the control group (**Figures 2C, D**; **Supplementary Figures S1A, B**). Notably, in the Cd+nSiO_2_ group, the levels of O_2_¯ and H_2_O_2_ in the root and leaf tissues were reduced by 31.68%, 39.9%, 39.69%, and 59.97%, respectively, compared with those in the Cd treatment alone group (**Figures 2C, D**; **Supplementary Figures S1A, B**).

### 3.3 Effect of nSiO_2_ on the antioxidant enzyme activity of pea seedlings under Cd stress

We subsequently investigated the activity of antioxidant system-related enzymes in pea seedlings subjected to Cd toxicity, nSiO_2_ exposure, or Cd+nSiO_2_ interactive treatment. These findings revealed that Cd triggered an increase in SOD activity in both the roots and leaves of pea plants. Specifically, compared with the control, Cd toxicity significantly increased SOD activity by 11.69% in pea seedling roots (**Figure 2E**). Conversely, when exposed to nSiO_2_ under Cd stress, SOD activity decreased by 5.30% compared with that under Cd treatment alone (**Figure 2E**). Similarly, compared with the control, Cd toxicity led to a significant increase of 23.71% in SOD activity in the leaves of pea seedlings (**Supplementary Figure S1C**). However, when exposed to nSiO_2_ under Cd stress, SOD activity was reduced by 9.88% compared with that under Cd alone (**Supplementary Figure S1C**).

On the other hand, compared with the control, exposure to Cd significantly increased the CAT activity in the roots and leaves of pea plants by 212.53% and 137.81%, respectively (**Figure 2F**; **Supplementary Figure S1D**). However, compared with the Cd treatment alone, the Cd+nSiO_2_ interaction treatment resulted in decreases in CAT activity of 54.12% and 30.15% in the roots and leaves of pea seedlings, respectively (**Figure 2F**; **Supplementary Figure S1D**). In addition, compared with the control, exposure to Cd significantly increased POD activity in the roots and leaves of pea plants by 26.71% and 87.57%, respectively (**Figure 2G**; **Supplementary Figure S1E**). However, compared with the Cd treatment alone, the Cd+nSiO_2_ interaction treatment resulted in decreases in POD activity of 14.23% and 28.98% in the roots and leaves of pea seedlings, respectively (**Figure 2G**; **Supplementary Figure S1E**).

### 3.4 Effect of nSiO_2_ on the cell membrane permeability of pea seedlings under Cd stress

The degree of oxidative damage and lipid peroxidation can be evaluated by the malondialdehyde (MDA) content in plants (Barclay and McKersie, 1994). We measured MDA levels in pea seedlings. Compared with that in the control plants, the content of MDA in the roots and leaves of pea plants significantly increased by 167.94% and 293.25%, respectively, under Cd stress (**Figure 2H**; **Supplementary Figure S1F**). Under Cd stress, the MDA content in the roots and leaves of pea seedlings treated with Cd+nSiO_2_ decreased significantly, by 30.07% and 60.90%, respectively, compared with that in the roots and leaves of pea seedlings treated with Cd alone (**Figure 2H**; **Supplementary Figure S1F**). Additionally, the relative electrical conductivity of pea seedlings can serve as an indicator of cell membrane permeability. Our results revealed that, compared with the control, Cd stress significantly increased the relative electrical conductivity of the roots and leaves of pea plants by 48.97% and 59.86%, respectively (**Figure 2I**; **Supplementary Figure S1G**). Compared with Cd treatment alone, treatment with Cd+nSiO_2_ resulted in a decrease in the relative electrical conductivity of pea seedling roots and leaves by 31.37% and 31.10%, respectively ((**Figure 2I**; **Supplementary Figure S1G**).

### 3.5 Effects of nSiO_2_ on the photosynthetic characteristics of pea seedlings under Cd stress

**Figure 3:**
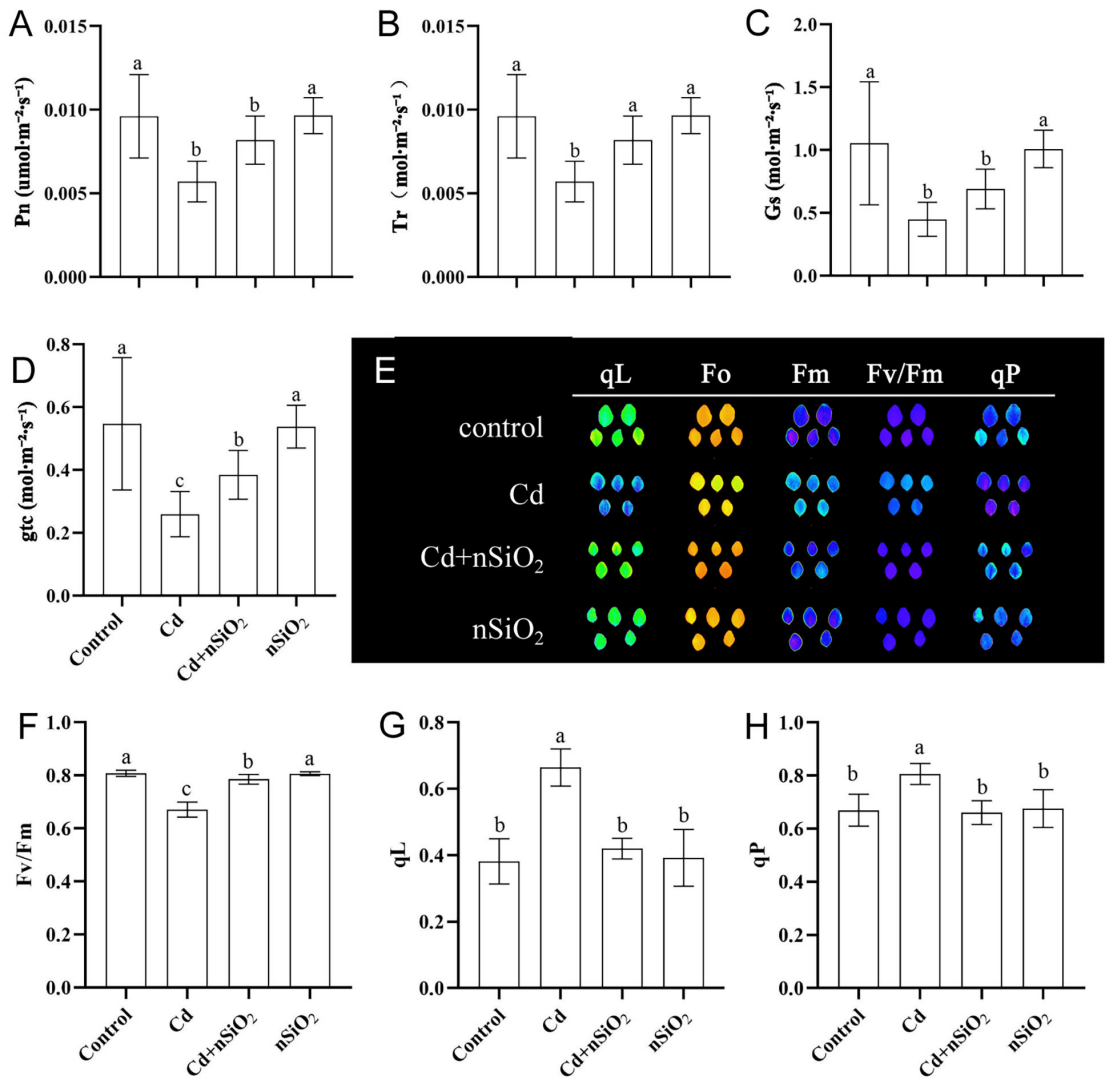
Nano-SiO_2_ exposure alleviated the inhibition of photosynthesis in pea plants induced by Cd. Ten-day-old germinated pea seedlings were transferred to 1/4-strength fresh Hoagland solution with or without 9 μM CdCl_2_, 50 mg/L nSiO_2_ (Control, Cd, Cd+nSiO_2_, nSiO_2_) for 12 days. The photosynthetic parameters **(A)** Pn, **(B)** Tr, **(C)** Gs and **(D)** gtc were measured. . **(E)** Representative chlorophyll fluorescence images and **(F–H)** quantification of **(F)** Fv/Fm, **(G)** qL and **(H)** qP. The results are presented as the means ± SDs (*n*=3). Different letters indicate significant differences (*P* < 0.05 according to Tukey’s test).

By observing the phenotype of pea plants, we found that Cd toxicity significantly impeded the growth and development of pea leaves (**Figure 1A**). We subsequently analyzed the relevant parameters of photosynthetic efficiency in pea seedlings subjected to Cd toxicity, nSiO_2_ exposure, or Cd+nSiO_2_ interactive treatment. The results indicated that a significant decrease in the photosynthetic rate of pea seedlings was affected by Cd toxicity (**Figures 3A–D**). Specifically, Cd toxicity led to substantial reductions of 45.23%, 40.63%, 57.39%, and 52.55% in the net photosynthetic rate (Pn), transpiration rate (Tr), stomatal conductance (Gs), and total conductance to CO_2_ (gtc), respectively, compared with those of the control (**Figures 3A–D**). However, under Cd stress, the combined treatment with Cd+nSiO_2_ increased the Pn, Tr, Gs, and gtc values by approximately 13.84%, 43.86%, 53.73%, and 48.31%, respectively, compared with those under Cd treatment alone (**Figures 3A–D**).

We then detected and analyzed the chlorophyll fluorescence-related parameters of the pea seedlings in each group. Compared with the control, Cd toxicity significantly reduced the maximal photosystem II (PSII) activity parameter (Fv/Fm) of pea seedlings by 16.93% (**Figures 3E, F**). Compared with the Cd alone treatment, the Cd+nSiO_2_ interaction treatment increased the Fv/Fm of the pea seedlings to 16.99% (**Figures 3E, F**). Conversely, compared with the control, Cd toxicity significantly increased the photochemical quenching coefficient (qP) and qL of pea seedlings by 20.37% and 73.98%, respectively (**Figures 3E, G, H**). Compared with Cd alone, the Cd+nSiO_2_ interaction treatment decreased the qP and qL of pea seedlings by 16.13% and 40.96%, respectively (**Figures 3E, G, H**).

### 3.6 Effects of nSiO_2_ on Cd and mineral element accumulation in pea seedlings under Cd stress

**Figure 4:**
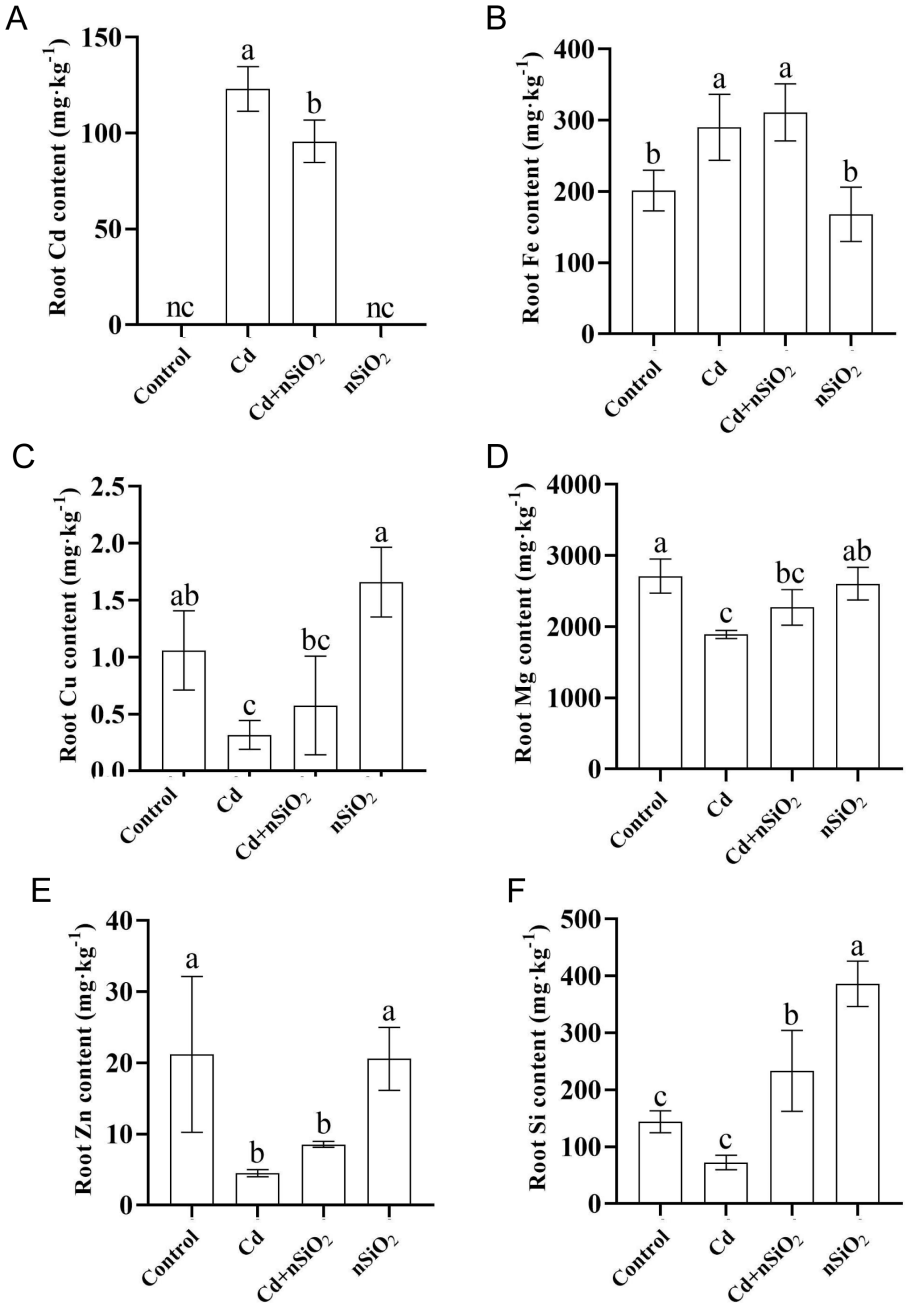
Nano-SiO_2_ exposure reduced Cd accumulation and improved the accumulation of mineral elements in pea roots under Cd stress. Ten-day-old germinated pea seedlings were transferred to 1/4-strength fresh Hoagland solution with or without 9 μM CdCl_2_, 50 mg/L nSiO_2_ (Control, Cd, Cd+nSiO_2_, nSiO_2_) for 12 days. The concentrations of **(A)** Cd, **(B)** Fe, **(C)** Cu, **(D)** Mg, **(E)** Zn and **(F)** Si were measured. The results are presented as the means ± SDs (*n*=3). Different letters indicate significant differences (*P* < 0.05 according to Tukey’s test).

The mineral element content of the pea seedlings was determined via inductively coupled plasma emission spectrometry (ICP–MS). Compared with Cd stress alone, the Cd+nSiO_2_ interaction treatment significantly reduced the Cd content in both the roots and leaves of pea seedlings by 22.24% and 67.88%, respectively (**Figure 4A**; **Supplementary Figure S2A**). All three independent biological replicates produced consistent results, confirming the reproducibility of the findings. Moreover, compared with the control, Cd toxicity increased the accumulation level of Fe in both the roots and leaves of pea plants by 44.06% and 42.51%, respectively (**Figure 4B**; **Supplementary Figure S2B**), with a significant increase observed specifically in the roots. However, there was no significant effect on Fe accumulation in pea seedlings subjected to the Cd+nSiO_2_ interaction treatment compared with those subjected to Cd toxicity alone (**Figure 4B**; **Supplementary Figure S2B**).

On the other hand, compared with the control, Cd toxicity significantly suppressed the accumulation of Cu, Mg, Zn, and Si in the roots of pea plants by 70.01%, 30.17%, 78.68%, and 49.55%, respectively (**Figures 4C–F**). Under Cd stress, the combined treatment of Cd+nSiO_2_ had no significant effect on the accumulation levels of Cu, Mg or Zn in pea seedlings (**Figures 4C–E**). Moreover, compared with the control, Cd toxicity inhibited the accumulation of Cu, Mg and Zn in the leaves of pea plants by 74.85%, 29.62%, and 54.05%, respectively (**Supplementary Figures S2C–E**). Under Cd stress, the Cd+nSiO_2_ interaction treatment promoted the accumulation of Cu, Mg and Zn in pea seedlings (**Supplementary Figures S2C–E**), but this difference did not reach statistical significance.

### 3.7 Transcriptome analysis

To explore the molecular mechanism underlying the alleviation of Cd toxicity to pea seedlings by nSiO_2_ and elucidate the potential regulatory pathway of the nSiO_2_-mediated response to Cd stress in pea, we performed transcriptome sequencing analysis on pea seedlings exposed to Cd toxicity, nSiO_2_ treatment, or Cd+nSiO_2_ interactive treatment. Each group of samples was subjected to three biological replicates, and the statistics of the filter quality control results are presented in **Supplementary Table S1**. After filtering the original sequencing data, we obtained a total of 151.26 Gb of clean data. The percentage of Q30 bases in all the transcriptome samples was 91.37% or greater, indicating acceptable data quality for each sample (**Supplementary Table S1**).

**Figure 5:**
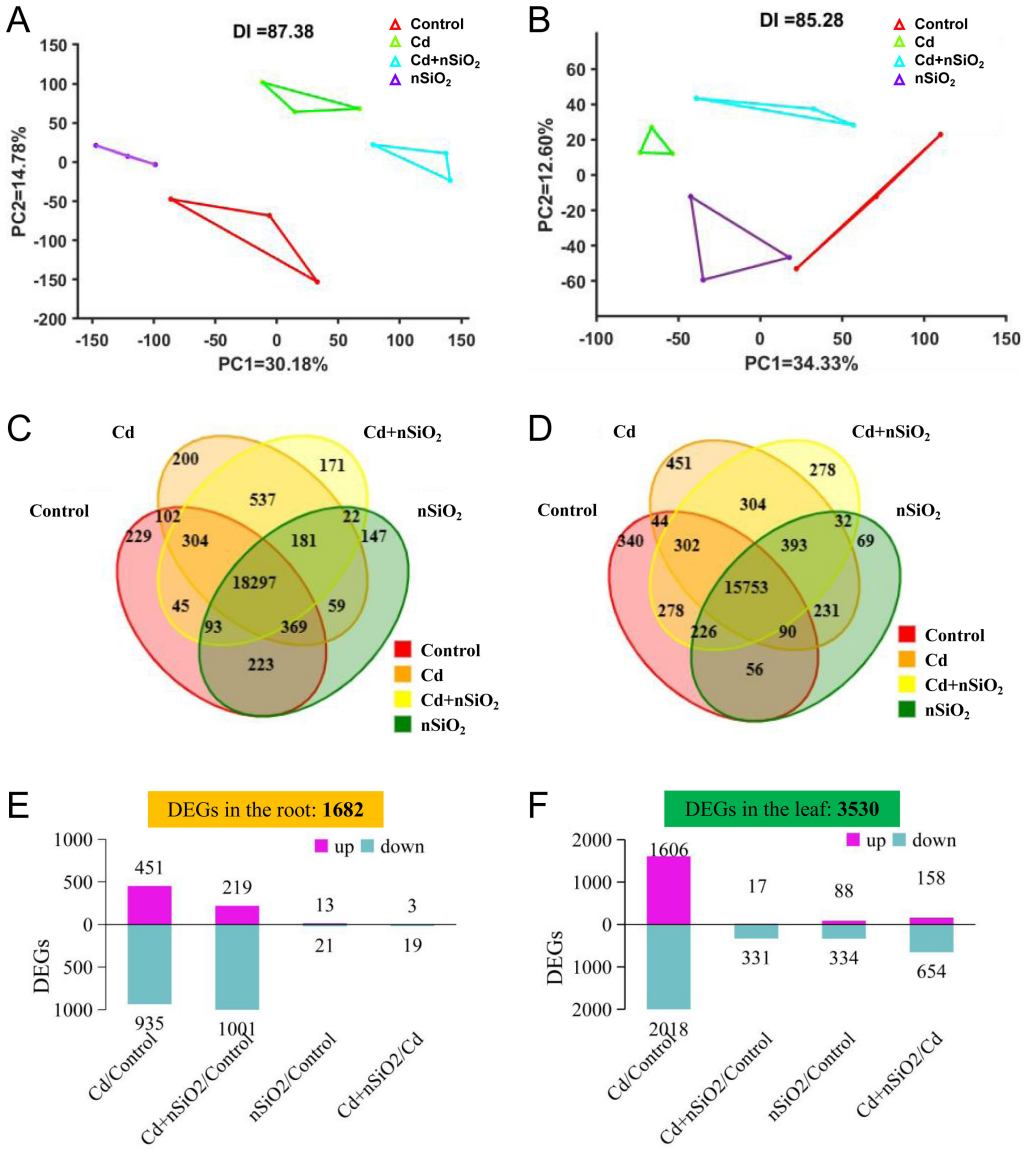
Transcriptome analysis of differentially expressed genes in pea seedlings treated with nSiO_2_ or Cd. Ten-day-old germinated pea seedlings were transferred to 1/4-strength fresh Hoagland solution with or without 9 μM CdCl_2_, 50 mg/L nSiO_2_ (Control, Cd, Cd+nSiO_2_, nSiO_2_) for 2 days. **(A, B)** Principal component analysis (PCA) score plots of pea **(A)** roots and **(B)** leaves. **(C, D)** Venn diagram analysis of differentially expressed genes (DEGs) in the **(C)** roots and **(D)** leaves of Control, Cd, nSiO_2_ and Cd+nSiO_2_ plants. **(E, F)** The number of upregulated and downregulated DEGs in the **(E)** roots and **(F)** leaves of the Cd/Control, nSiO_2_/Control, Cd+nSiO_2_/Control and Cd+nSiO_2_/Cd comparisons.

We then generated an FPKM box plot to visualize the gene expression of each transcriptome sample (**Supplementary Figure S3**). The discrete distribution of gene expression in the transcriptome samples of each group directly reflects the overall gene expression of different samples (**Supplementary Figure S3**). Additionally, we evaluated the dispersion of samples within each treatment group via principal component analysis (PCA). In **Figures 5A, B**, PC1 on the x-axis represents the first principal component, with its percentage indicating its contribution to sample differences. The contribution values of the first principal component in the roots and leaves of the pea seedlings to the sample difference were 30.18% and 34.33%, respectively (**Figures 5A, B**). On the y-axis, PC2 represents the second principal component. The contribution values of the second principal component in the roots and leaves of the pea seedlings to the sample difference were 14.70% and 12.60%, respectively (**Figures 5A, B**). These results indicate that the similarity between the transcriptome repeats is high, which meets the requirements of transcriptome data analysis and can be used for subsequent differentially expressed genes (DEGs) analysis.

### 3.8 Analysis of DEGs in pea seedlings in each group

The Venn diagram provides a visual representation of the common and unique genes detected in each transcriptome sample. **Figures 5C, D** show Venn diagrams depicting gene detection between four treated transcriptome samples from pea roots and leaves, respectively. A total of 20,979 genes were detected in pea roots (**Figure 5C**). Among these genes, 18,297 coexpressed genes were unrelated to exogenous treatment. Additionally, 908 genes whose expression was specifically associated with only Cd toxicity (the Cd toxicity and Cd+nSiO_2_ treatment groups) were identified. Furthermore, 171 genes were specifically expressed in response to the Cd+nSiO_2_ treatment (**Figure 5C**). Similarly, a total of 18,847 genes were detected in pea leaves (**Figure 5D**). Among these genes, 15,753 coexpressed genes were not related to exogenous treatment. Moreover, there were 1,033 genes whose expression was exclusively associated with Cd toxicity (the Cd toxicity and Cd+nSiO_2_ treatment groups). Finally, 278 genes were specifically expressed in response to the Cd+nSiO_2_ treatment (**Figure 5D**).

The DEGs (|log2FC|≥1 and q value ≤ 0.05) were subsequently subjected to further analysis among the various pea seedling treatment groups. Comparative analysis revealed 1682 and 3530 DEGs in the roots and leaves of pea seedlings, respectively (**Figures 5E, F**). Compared with those in the control, a total of 1386 DEGs (including 451 upregulated and 935 downregulated) and 3624 DEGs (including 1606 upregulated and 2018 downregulated) were identified in the roots and leaves of pea seedlings under Cd toxicity (Cd/control), respectively (**Figures 5E, F**). In addition, 34 DEGs (including 13 upregulated DEGs and 21 downregulated DEGs) and 422 DEGs (including 88 upregulated DEGs and 334 downregulated DEGs) were identified in the roots and leaves of pea seedlings treated with nSiO_2_ (nSiO_2_/control) (**Figures 5E, F**). A total of 1220 DEGs (including 219 upregulated and 1001 downregulated) and 348 DEGs (including 17 upregulated and 331 downregulated) were identified in the roots and leaves of pea seedlings treated with Cd+nSiO_2_ (Cd+nSiO_2_/Control), respectively (**Figures 5E, F**). In addition, compared with those under the Cd treatment alone, 22 DEGs (3 upregulated and 19 downregulated) and 812 DEGs (158 upregulated and 654 downregulated) were identified in the roots and leaves of peas under the Cd+nSiO_2_ treatment (Cd+nSiO_2_/Cd), respectively (**Figures 5E, F**).

### 3.9 DEGs cluster analysis of pea seedlings subjected to Cd toxicity and nSiO_2_ exposure

**Figure 6:**
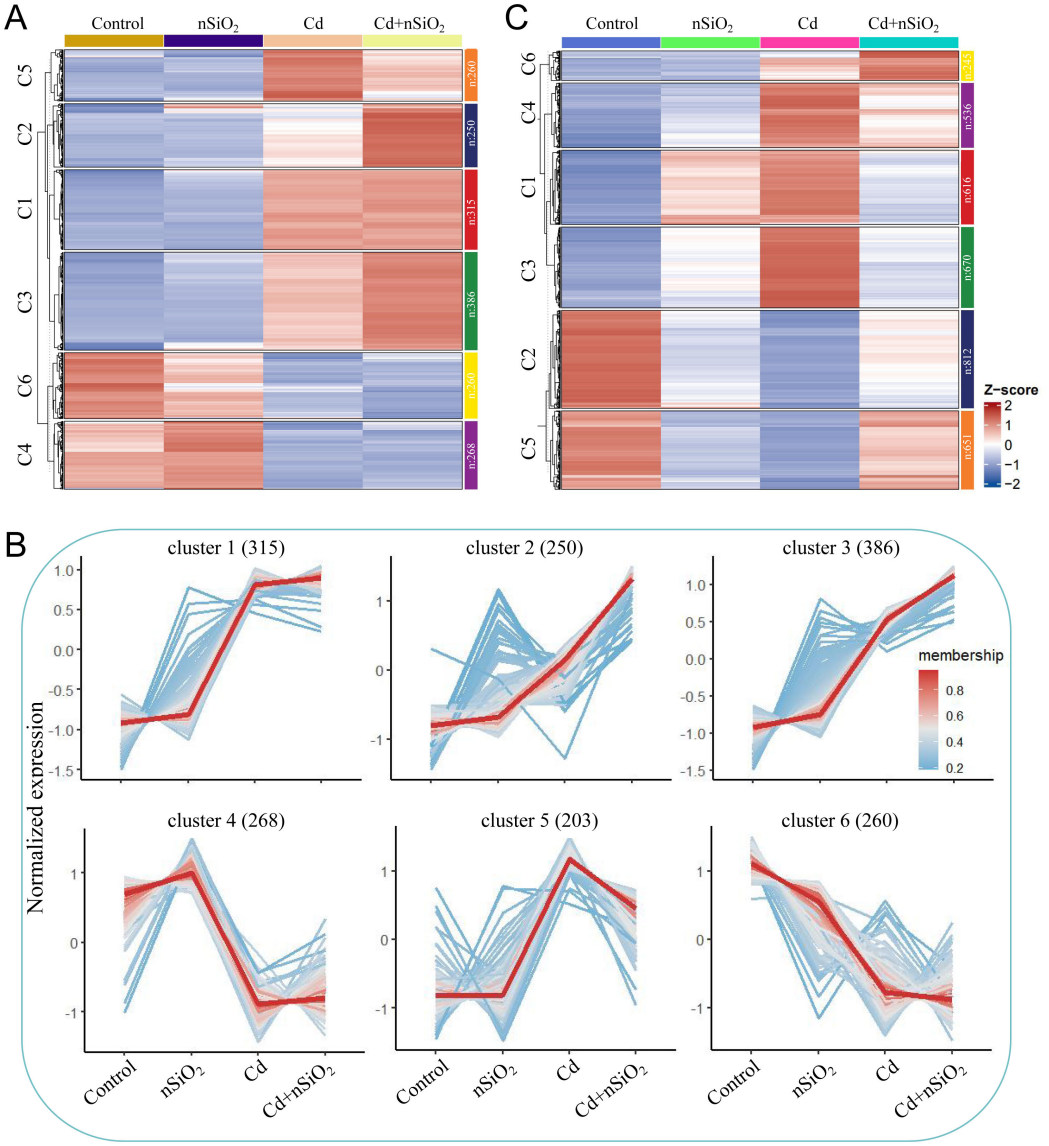
Perform cluster analysis on DEGs identified in the samples of four groups of pea seedlings under different treatments. Cluster analysis was performed on four groups of DEGs identified under different treatments in pea **(A)** roots and **(C)** leaves. **(B)** Analysis of gene expression trend under different treatments in gene clusters in pea seedlings roots.

We subsequently used the K-means algorithm to perform cluster analysis on 1682 DEGs identified in the root samples of the four groups of pea seedlings under different treatments. The clustering results revealed that these DEGs were clustered into 6 gene clusters (**Figure 6A**). The number of DEGs in cluster 1, cluster 2, cluster 3, cluster 4, cluster 5 and cluster 6 in the roots of the pea seedlings was 315, 250, 386, 268, 203 and 260, respectively (**Figure 6A**). As shown in **Figure 6B**, Cd toxicity significantly induced gene expression in cluster 1, cluster 2, cluster 3, and cluster 5 in the roots of pea seedlings. In contrast, Cd toxicity significantly inhibited gene expression in Clusters 4 and 6 (**Figures 6A, B**). Further analysis revealed that the expression of genes in cluster 4 slightly increased under nSiO_2_ exposure, significantly decreased under Cd toxicity, and slightly increased under the Cd+nSiO_2_ interactive treatment (compared with that under Cd toxicity alone) (**Figure 6B**). The expression trends of these genes were consistent with the growth and development phenotypes of the pea seedlings in the four treatment groups (**Figure 6B**; **Figures 1A–G**). On the other hand, the expression of genes in cluster 5 essentially remained unchanged under nSiO_2_ exposure, significantly increased under Cd toxicity, and significantly decreased under the Cd+nSiO_2_ interactive treatment (compared with that under Cd toxicity alone) (**Figure 6B**). The expression trends of these genes were opposite those of the growth and development phenotypes of the pea seedlings in the four treatment groups (**Figure 6B**; **Figures 1A–G**).

On the other hand, we used the K-means algorithm to perform cluster analysis on 3530 DEGs identified from pea seedling leaf samples in the four treatment groups. The clustering results revealed that these DEGs were clustered into 6 gene clusters (**Figure 6C**). The number of DEGs in cluster 1, cluster 2, cluster 3, cluster 4, cluster 5 and cluster 6 in the leaves of pea seedlings was 616, 812, 670, 536, 651 and 245, respectively (**Figure 6C**). As shown in **Supplementary Figure S4**, Cd toxicity significantly induced gene expression in cluster 1, cluster 3, cluster 4 and cluster 6 pea leaves. In contrast, Cd toxicity significantly inhibited gene expression in cluster 2 and cluster 5 (**Supplementary Figure S4**). Further analysis revealed that the expression of genes in clusters 2 and 5 significantly decreased under nSiO_2_ exposure, significantly decreased under Cd toxicity, and significantly increased under the Cd+nSiO_2_ interactive treatment (compared with that under Cd toxicity alone) (**Supplementary Figure S4**). The change trend of DEG expression was similar to the change trend of the growth and development phenotypes of pea seedlings in the four treatment groups (**Supplementary Figure S4**; **Figures 1A–G**). In contrast, the expression of genes in cluster 3 and cluster 4 increased under nSiO_2_ exposure, significantly increased under Cd toxicity, and significantly decreased under the Cd+nSiO_2_ interactive treatment (compared with that under Cd toxicity alone) (**Supplementary Figure S4**). The change trend of DEG expression contrasted with the change trend of the growth and development phenotypes of pea seedlings in each treatment group (**Supplementary Figure S4**; **Figures 1A–G**).

### 3.10 KEGG enrichment analysis of DEGs in pea seedlings subjected to Cd toxicity and nSiO_2_

**Figure 7:**
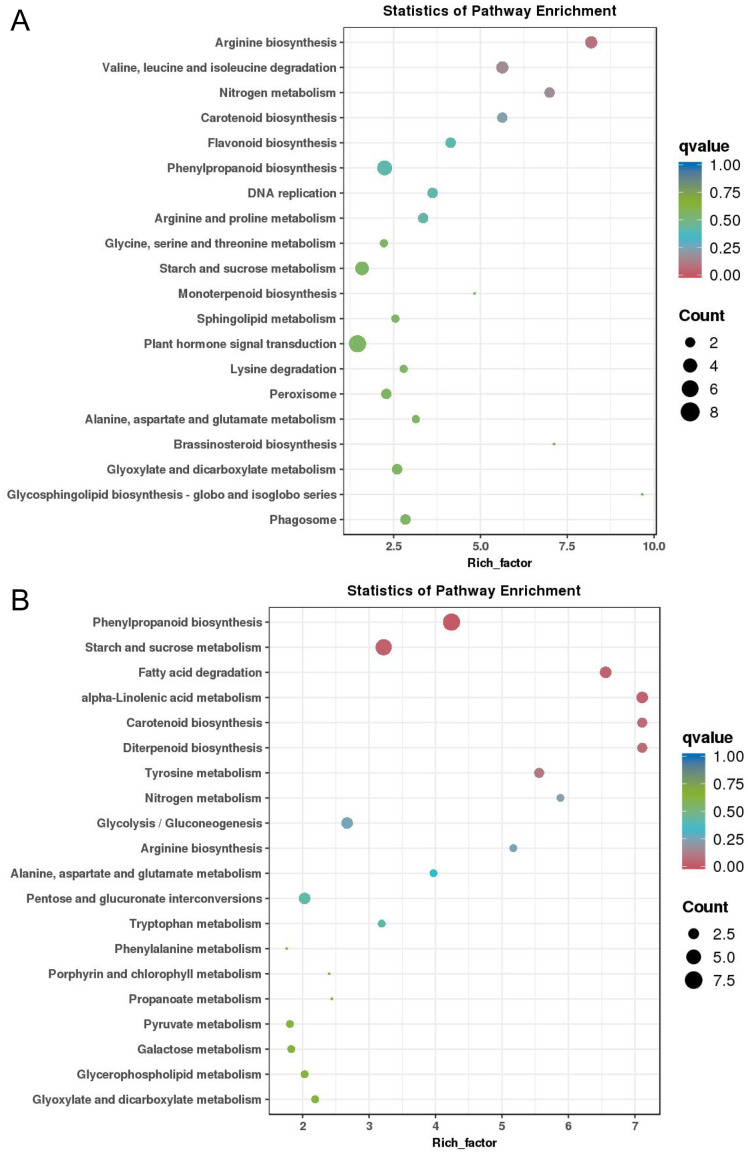
KEGG enrichment analysis of the differentially expressed genes in pea seedlings. Top 20 enriched KEGG pathways of DEGs from **(A)** cluster 4 and **(B)** cluster 5 in pea seedling roots.

On the basis of the above results, KEGG enrichment analysis was performed on 268 DEGs from cluster 4 in pea seedling roots (**Figure 6C**; **Figure 7A**). The results revealed that the DEGs in root cluster 4 were significantly enriched in alanine, aspartate and glutamate metabolism; arginine biosynthesis; the phenylpropanoid biosynthesis pathway; etc. (**Figure 7A**). The 203 DEGs in cluster 5 of pea seedling roots were significantly enriched in starch and sucrose metabolism, carotenoid biosynthesis, nitrogen metabolism, arginine biosynthesis and other pathways (**Figure 7B**). On the other hand, KEGG enrichment analysis was performed on 670 DEGs from cluster 3 in pea seedling leaves (**Supplementary Figure S4**; **Supplementary Figure S5A**). The results revealed that DEGs in leaf cluster 3 of pea seedlings were significantly enriched in the biosynthesis of amino acids, starch and sucrose metabolism, fatty acid metabolism, and the flavonoid biosynthesis pathway (**Supplementary Figure S5A**). The 536 DEGs in cluster 4 of pea seedling leaves were significantly enriched in phenylpropanoid biosynthesis, starch and sucrose metabolism, amino acid biosynthesis, endocytosis and other pathways (**Supplementary Figure S5B**).

**Figure 8:**
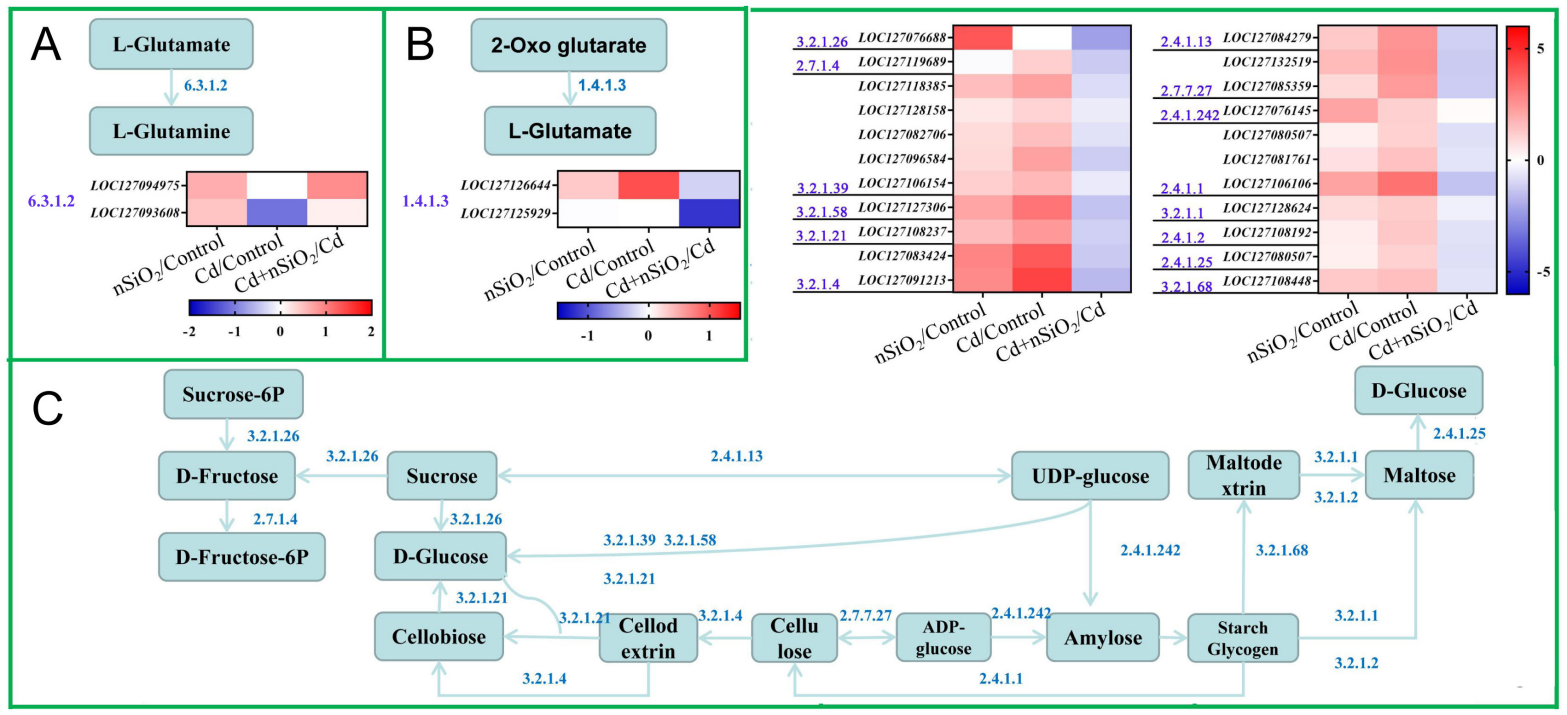
Metabolic pathway analysis of DEGs enrichment in the roots and leaves of pea seedlings. **(A)** Alanine, aspartate and glutamate metabolism pathways analysis in pea seedling root cluster 4. **(B)** Alanine, aspartate and glutamate metabolism pathways analysis in pea seedling root cluster 5. **(C)** Starch and sucrose metabolism pathways analysis in pea seedling leaf cluster 3.

Further analysis revealed that the *LOC127094975* and *LOC127093608* genes were enriched in the alanine, aspartate and glutamate metabolism pathways in pea seedling root cluster 4. These proteins play important regulatory roles in the conversion of L-glutamate to L-glutamine (**Figure 8A**). The heatmap shows that the expression of these genes is reduced under Cd stress, thus inhibiting this metabolic process. However, the expression of these genes in the roots of peas increased after exposure to nSiO_2_ under Cd stress (**Figure 8A**). The expression trends of these genes were consistent with the growth and development phenotypes of the pea seedlings in the four treatment groups (**Figure 8A**; **Figures 1A–G**). In root cluster 5 of pea seedlings, two genes, *LOC127126644* and *LOC127125929*, were significantly enriched in the alanine, aspartate and glutamate metabolism pathways (**Figure 8B**). They play crucial roles in regulating the conversion of 2-oxoglutamate to L-glutamate. The heatmap clearly shows that the expression levels of these genes increase under Cd stress, thereby facilitating this metabolic process. Conversely, exposure to nSiO_2_ under Cd stress led to a decrease in the expression levels of these genes in pea roots (**Figure 8B**). The pattern of gene expression changes contrasted with the trend of growth and developmental phenotypic changes in pea seedlings under the four treatments (**Figure 8B**; **Figures 1A–G**).

On the basis of these previous results, KEGG enrichment analysis was performed on 670 DEGs from Cluster 3 in the leaves of pea seedlings (**Supplementary Figure S4A**). The results revealed that the DEGs in cluster 3 of pea seedling roots were significantly enriched in the ribosome, biosynthesis of amino acids, anthocyanin biosynthesis, starch and sucrose metabolism, flavonoid biosynthesis and other pathways (**Supplementary Figure S4A**). A total of 536 DEGs from cluster 4 pea seedling leaves were significantly enriched in glutathione metabolism, phenylpropanoid biosynthesis, starch and sucrose metabolism, and the endocytosis pathway (**Supplementary Figure S4B**).

Further analysis revealed that the genes enriched in the starch and sucrose metabolism pathways in leaf cluster 3 of pea seedlings were as follows: *LOC127076145*, *LOC127108237*, *LOC127081761*, *LOC127118385*, *LOC127119689*, *LOC127132519*, *LOC127127306*, *LOC127128158*, *LOC127128624*, *LOC127076688*, *LOC127082706*, *LOC127085359*, *LOC127080507*, *LOC127083424*, *LOC127084279*, *LOC127091213*, *LOC127096584*, *LOC127108448*, *LOC127 106106*, *LOC127106154* and *LOC127108192* (**Figure 8C**). These genes encode granule-bound starch synthase 1, B-S glucosidase 44, alpha-glucan phosphorylase 2, O-glycosyl hydrolase family 17 protein, fructokinase-like 2, metal ion-binding protein, endo-beta-mannase 5, O-glycosyl hydrolase family 17 protein, alpha-amylase-like 3,6-fructan exohydrolase, O-glycosyl hydrolase family 17 protein, APL4, disproportionate enzyme, glycosyl hydrolase 9B13, sucrose synthase 6, glycosyl hydrolase 9B13, carbohydrate-binding X8 domain superfamily protein, isoamylase 1, alpha-glucan phosphorylase 1, plasasmaodesmata callose-binding protein 5, and reduced beta amylase 1, respectively. These genes regulate the conversion pathway of sucrose to fructose and glucose (**Figure 8C**). The expression of these genes increased under Cd stress, thus promoting this metabolic process. Under Cd stress, nSiO_2_ exposure decreased the expression levels of these genes in pea leaves. The expression trend of these genes was opposite to that of the growth and development phenotypes of pea seedlings in the four groups (**Figure 8C**; **Figures 1A–G**).

## 4 Discussion

Cd, a nonessential element in organisms, exhibits strong biological toxicity and high environmental migration characteristics, facilitating its absorption and accumulation by crops (Franić and Galić, 2019; Shaari et al., 2022; Sterckeman and Thomine, 2020; Hussain et al., 2021). The excessive presence of Cd in plants leads to oxidative stress and membrane lipid peroxidation, disrupts photosynthetic functions, and diminishes photosynthesis, ultimately impeding plant growth and reducing biomass (Zhao et al., 2021; Benavides et al., 2005). Adsorption is widely regarded as a highly promising technology for mitigating environmental pollutants, offering significant advantages such as cost-effectiveness, operational simplicity, and low energy consumption. Among various adsorbents, porous nanomaterials have shown exceptional performance and play an important role in the removal of contaminants such as heavy metals (Ye et al., 2023). Furthermore, Pu et al. (2019) investigated the effect of copper oxide nanoparticles (nCuO) on copper uptake in maize and observed that nCuO could supplement copper nutrition in plants. De Sousa et al. (2019) demonstrated that nSiO_2_ can ameliorate the phytotoxic effects of Al in maize cultivated in acidic soil. This suggests that metal oxide nanoparticles are capable of dissociating to some extent, releasing metal ions. Previous studies have indicated that the uptake of mineral elements in plants involves competition among metal ions for transport via metal ion channel proteins (Inglezakis et al., 2005). Therefore, metal oxide nanoparticles may inhibit the uptake of heavy metals in plants through a competitive mechanism mediated by the ions they release.

Our study demonstrated that exposure to nSiO_2_ effectively mitigated the oxidative damage induced by Cd toxicity (**Figure 2**), attenuated its adverse impact on photosynthetic efficiency (**Figure 3**), enhanced mineral nutrient uptake under Cd stress (**Figure 4**), and promoted pea growth (**Figure 1**). Research has shown that Cd stress adversely affects plant height, leaf area, and biomass in *Indian mustard* and saffron seedlings (Jiang et al., 2004; Zhao et al., 2021). Furthermore, nSiO_2_ promotes plant growth, enhances development, and increases plant stress resistance. Studies have shown that nSiO_2_ alleviates seed germination under heavy metal stress. For example, Sun et al. (2023) revealed that nSiO_2_ significantly mitigated the inhibitory effects of Cd toxicity on *Momordica balsam* seedling growth. Additionally, nSiO_2_ reduces Cd absorption, modulates nutrient balance, and regulates the antioxidant enzyme system in barley seedlings under Cd stress (He et al., 2023). Emamverdian et al. (2020) reported that in the presence of heavy metals (Cu and Mn), nSiO_2_ enhanced protective enzyme activity, chlorophyll content, and photosynthetic efficiency, leading to increased biomass, stem length, and overall plant growth while reducing Cu and Mn toxicity. Our experiment also revealed that nSiO_2_ significantly improved the pea seed germination rate and potential under Cd stress, highlighting its ability to alleviate Cd toxicity during pea seed germination (**Figure 1**), possibly through mechanisms akin to those described by Emamverdian et al. (2020).

When plants undergo stress, the levels of ROS in their systems increase rapidly. The excessive presence of O_2_¯ and H_2_O_2_ results in oxidative stress, damaging plant membranes. SOD, POD, CAT, and other antioxidant enzymes play critical roles in the antioxidant defense system of plants. In response to stress, the activities of SOD, POD, and CAT increase to efficiently eliminate ROS and maintain normal metabolic equilibrium. Leng et al. (2021) reported that Cd significantly increased the activity of APX and CAT in the roots and stems of mung bean seedlings. On the other hand, Dong et al. (2016) reported that peanuts have the ability to mitigate Cd toxicity by increasing the activities of antioxidant enzymes (POD and CAT). Additionally, they reported a positive relationship between the Cd concentration and enzyme activity. Moreover, it increased POD activity in the roots, stems, and leaves while increasing the levels of leaf chlorophyll, carotenoids, root polyphenols, MDA, and proline. Conversely, the activity of CAT in leaves, the levels of AsA in roots and leaves, and the levels of polyphenols in stems and leaves decreased. Similarly, *Perilla* seedlings exposed to Cd stress presented elevated levels of O_2_¯ and MDA and increased antioxidant enzyme activity, in addition to reduced biomass and root dry weight (Wang et al., 2022). This study of pea seedlings subjected to Cd stress revealed notable increases in ROS (O_2_¯, H_2_O_2_) and MDA contents, accompanied by increased activities of antioxidant enzymes (SOD, POD, and CAT). Consequently, reduced membrane lipid peroxidation hinders root function, leading to inhibited plant growth (**Figure 1**; **Figure 2**; **Supplementary Figure S1**). However, when the pea seedlings were exposed to nSiO_2_ under Cd stress, the activities of the antioxidant enzymes SOD, POD, and CAT decreased, as did the levels of O_2_¯, H_2_O_2_, and MDA (**Figure 1**; **Figure 2**; **Supplementary Figure S1**). These results indicate that nSiO_2_ can mitigate the oxidative stress induced by Cd toxicity, thereby alleviating its inhibitory impact on the growth of pea seedlings.

The accumulation of Cd in roots caused significant phytotoxicity in plants. Studies by Muhammad Arshad et al. (2016) have demonstrated that Cd stress inhibits gas exchange in wheat seedlings, exacerbating the connection between photosynthesis and gas exchange. Additionally, Cd triggers the degradation of chlorophyll, resulting in a loss of photosynthetic pigments (Sun et al., 2023). Disruption of PSII by Cd leads to a decrease in initial fluorescence (F_0_) and Fv/Fm in *Solanum* leaves (Gharbi et al., 2018). Janeeshma et al. (2021) reported that Cd stress significantly impacts early photochemical reactions in maize. Research has also revealed that the nonspecific toxic effects of Cd cause chlorophyll degradation, decrease the efficiency of PSII, and interfere with electron transport processes. Farhat et al. (2022) reported notable reductions in the Fv/Fm, qP, electron transport rate (ETR), and gas exchange properties of wheat under Cd stress. They also noted that Cd-induced plant toxicity increased nonphotochemical quenching (NPQ) and the internal carbon dioxide concentration (Ci). Our study focused on the chlorophyll fluorescence parameters of pea seedlings under Cd stress and revealed a decrease in the photochemical quenching coefficient of the leaves, the Fv/Fm of PSII, and the Pn and Tr (**Figure 3**). These findings imply that Cd inhibits photosynthetic efficiency by suppressing photochemical reactions. The inhibition of photosynthetic characteristics by Cd, such as reduced chlorophyll content, impaired photochemical reactions, gas exchange, and transpiration rates, is widely recognized as the primary cause of plant growth inhibition (Aqeel et al., 2021). Interestingly, the application of nSiO_2_ to pea seedlings under Cd toxicity resulted in a decrease in the photochemical quenching coefficient of the leaves but increased the Fv/Fm, Pn and Tr of the seedling leaves (**Figure 3**). These results suggest that nSiO_2_ can alleviate the inhibitory effects of Cd toxicity on pea seedling growth by restoring photochemical reactions.

The absorption of nutrients in plants is hampered by Cd due to the lack of a specific transporter for Cd in plants. As a result, Cd competes with essential cations such as Ca^2+^, Cu^2+^, Zn^2+^, and Mg^2+^ for transmembrane transport (Pinto and Ferreira, 2015), leading to an imbalance of mineral elements within plants. Magnesium plays a pivotal role as a central metal element with chelating effects at the core of the chlorophyll porphyrin ring, and its deficiency can negatively impact chlorophyll synthesis in plants (Hansson et al., 2013). Copper is predominantly found in chloroplasts, and a deficiency in copper can disrupt the stability of chlorophyll in leaves. Zinc acts as a catalyst during chlorophyll synthesis, and its deficiency can reduce the transpiration rate and stress resistance of plants (Rodríguez et al., 2018). Iron not only aids in chlorophyll synthesis but also supports photosynthesis and respiration processes as a cofactor for various functional proteins in plants. Within photosystem I, iron combines with sulfur to form ferrithioreducin, which actively participates in electron transport (Sticht and Rösch, 1998). Our investigation revealed that Cd toxicity significantly hindered the uptake of Mg, Cu, and Zn in both the roots and leaves of pea (**Figures 4C–E**; **Supplementary Figure S2**). This disruption in elemental balance leads to compromised chlorophyll synthesis and stability, damage to the chloroplast structure, interference with the Q cycle, and accumulation in the initial reaction of PSII, ultimately inhibiting plant photosynthesis (de Bang et al., 2021). Notably, the application of nSiO_2_ pea seedlings under Cd toxicity promoted the absorption of Mg, Cu, and Zn. Consequently, nSiO_2_ alleviated the inhibitory effects of Cd on pea photosynthesis efficiency (**Figures 4C–E**; **Figure 3**). These results indicate the potential of nSiO_2_ to increase pea photosynthesis efficiency and alleviate Cd-induced growth inhibition by facilitating the absorption of mineral elements.

Our previous research revealed that Cd toxicity inhibits the growth of tomato seedlings by disrupting metabolic pathways involving arginine, proline, alanine, aspartic acid, and glutamic acid (Sun et al., 2023). Phenylalanine and tyrosine play a role in the synthesis of cinnamic acid and its hydroxyl derivatives (Jitareanu et al., 2011), which act as precursors for lignin production, polyphenols, and their derivatives while also regulating various physiological processes in plants (Shuab et al., 2016). In this study, six clusters of DEGs were identified from pea seedling roots and leaves through clustering analysis. KEGG enrichment analysis was subsequently conducted to identify prominent Cd-responsive metabolic regulatory pathways (**Figure 6**; **Figure 7**; **Supplementary Figure S4**). Notably, the gene expression changes in pea seedling root cluster 4 were consistent with the growth and development phenotypes of pea seedlings under different treatments, whereas the changes in cluster 5 were inversely related to the growth and development phenotypes under the four treatments (**Figure 6B**; **Figures 1A–G**). In the alanine, aspartate, and glutamate metabolism pathway (Root Cluster 4), *LOC127094975* and *LOC127093608* play crucial roles in the conversion of L-glutamate to L-glutamine. Their downregulation under Cd stress and recovery under nSiO_2_ + Cd treatment correlate with restored growth phenotypes (**Figure 8A**). Cd toxicity led to the suppression of *LOC127093608* expression, causing the accumulation of L-glutamate. Conversely, the genes *LOC127126644* and *LOC127125929* were enriched in the same pathway within cluster 5 of pea seedling roots, with a significant role in the conversion of 2-oxoglutarate to L-glutamate (**Figure 8B**). Cd toxicity resulted in the upregulation of *LOC127126644* expression, leading to the accumulation of L-glutamate. These findings suggest that the accumulation of L-glutamate, regulated by the genes *LOC127094975*, *LOC127093608*, *LOC127126644*, and *LOC127125929*, plays a pivotal role in the response of peas to Cd toxicity. Under Cd-induced stress, exposure to nSiO_2_ influenced the expression levels of these genes in pea roots (**Figures 8A, B**), indicating their potential as candidate genes for the nSiO_2_-mediated response to Cd stress in peas.

Under Cd stress, plant cells actively participate in the perception of Cd and subsequent response mechanisms by activating defence-related genes through pathways such as calcium signaling and phytohormone signaling (Al-Khayri et al., 2023). The interdependent metabolic and transport processes of carbon (C) and nitrogen (N) play a regulatory role in plant growth, development, and stress responses. Yin et al. (2024) found that the negative impact of Cd stress on defense gene expression and metabolic profiles in *Salix viminalis* was counteracted by melatonin, which restored homeostasis in leaf amino acid and carbohydrate metabolic pathways. In rice, the transcription factor Nhd1 modulates these processes by directly activating the expression of *OsSUT1* (sucrose transporter 1), which in turn leads to extensive reprogramming of gene expression involved in starch, sucrose, and amino acid metabolic pathways (Li et al., 2023). The metabolism of sucrose is crucial for plant growth and development, as it not only serves as an energy source and a structural component but also functions as an antioxidant to increase plant resilience against external stresses (Granot et al., 2013). Yokotani et al. (2009) demonstrated that the transcription factor ONAC063 in rice can upregulate salt stress response genes and the amylase gene *AMY1* in *Arabidopsis*, indicating its regulatory role in salt stress. In starch and sucrose metabolism (Leaf Cluster 3), we identified genes such as *LOC127076145* (granule-bound starch synthase 1) and *LOC127128624* (amylase-like protein), which are involved in starch degradation and sucrose conversion. Their expression patterns under different treatments are explicitly linked to carbon allocation and energy supply under stress. Notably, *LOC127128624* plays a significant role in the Cd stress response in pea plants (**Figure 8C**). Furthermore, Zhai et al. (2021) reported that ectopic expression of *OLEOSIN 1* and inactivation of the starch synthase gene *GBSS1* had synergistic effects on lipid accumulation in plant leaves. Here, we found that the *LOC127076145* gene in pea leaves encodes granule-bound starch synthase 1, which is highly expressed under Cd stress (**Figure 8C**), indicating a potential increase in starch accumulation in response to Cd toxicity. Additionally, we observed that the Cd stress response gene *LOC127081761* in peas facilitates the conversion of starch glycogen to cellulose (**Figure 8C**), suggesting that it plays a role in enhancing plant Cd tolerance by regulating cell wall formation. Transcriptome analysis revealed opposite expression patterns of these DEGs in peas under the Cd+nSiO_2_ interaction treatment compared with those under Cd toxicity alone (**Figure 8C**), indicating that these DEGs could be candidate genes for the nSiO_2_-mediated Cd stress response in peas. However, further exploration is needed to understand the functions and regulatory mechanisms of these DEGs.

In this study, we investigated the physiological and molecular mechanisms underlying nSiO_2_-induced alleviation of Cd toxicity in pea seedlings by evaluating growth parameters, oxidative stress, photosynthetic efficiency, mineral nutrient uptake, and transcriptomic profiles. Our integrated data provide compelling indirect evidence that nSiO_2_ application reduces Cd uptake and alleviates its phytotoxicity, likely through mechanisms such as surface complexation, ion exchange, or co-precipitation—consistent with previous reports (Cui et al., 2017; Sun et al., 2023). The decreased Cd accumulation in roots and leaves following nSiO_2_ treatment (**Figure 4A**) further supports the presence of adsorption-based interactions between nSiO_2_ and Cd ions. Prior studies have confirmed that nanoparticles can bind Cd²^+^ via surface complexation and ion exchange (Cui et al., 2017; Rizwan et al., 2019), thereby reducing metal bioavailability. Beyond adsorption, our transcriptomic analysis revealed that nSiO_2_ modulates pivotal metabolic pathways in pea plants, especially in sucrose and amino acid metabolism, enhancing internal detoxification capability. These findings expand the current mechanistic understanding of nanoparticle-mediated mitigation of Cd stress. Furthermore, in contrast to monocot species such as rice and maize, legumes exhibit distinct regulatory responses in nutrient metabolism, underscoring the importance of species-specific mechanisms in nanoparticle-assisted phytoremediation. Our results highlight the dual role of nSiO_2_ in not only adsorbing heavy metal ions but also reprogramming physiological processes, supporting its potential application in sustainable agriculture within contaminated environments.

Notwithstanding these insights, the current study acknowledges certain limitations. The contribution of adsorption mechanisms to nSiO_2_-mediated Cd mitigation remains incompletely characterized and warrants further investigation. While our study demonstrates nSiO_2_-mediated reduction in Cd uptake through physiological and transcriptomic evidence, future incorporation of adsorption models such as Langmuir and Freundlich isotherms could quantitatively characterize binding capacity and affinity between nSiO_2_ and Cd²^+^. Such modeling would provide crucial parameters to complement the molecular mechanisms identified here and further bridge nanoparticle surface interactions with plant physiological responses. Future studies should therefore employ dedicated adsorption experiments, including time-dependent kinetic assays and equilibrium isotherm analyses, complemented by robust statistical validation, to rigorously quantify nSiO_2_ adsorption behavior. Established models in adsorption science—such as Langmuir, Freundlich, and pseudo-second-order kinetics—provide standardized frameworks for quantifying adsorption capacity and dynamics. Applying these modeling approaches will enable systematic characterization of nSiO_2_–Cd²^+^ interactions, allow direct comparisons with other nano-agricultural systems, and yield deeper mechanistic insights into the adsorption process.

## 5 Conclusion

Cd is one of the most toxic heavy metals to organisms, and its pollution poses a threat to the sustainable development of agriculture and food safety (Godt et al., 2006; Kaur et al., 2018). This study investigated the impact of nSiO_2_ on pea growth under Cd-induced stress. Exposure to nSiO_2_ enhances pea seedling resistance to oxidative stress, increases mineral element accumulation, and improves photosynthetic efficiency, consequently fostering pea seedling growth under Cd stress. Analysis at the transcriptomic level revealed that nSiO_2_ altered C/N metabolic pathways, particularly sucrose and amino acid metabolism, in pea seedlings. Furthermore, the expression profiles of DEGs associated with these metabolic pathways were significantly correlated with plant growth and development. Nevertheless, certain limitations should be considered in this study. The experiments were conducted under controlled hydroponic conditions, which may not fully reflect the complex soil-plant interactions occurring in natural field environments. Factors such as soil organic matter, microbial activity, and variable climatic conditions could significantly influence the efficacy and behavior of nSiO_2_ in practical applications. The current study lacks detailed adsorption kinetics and isotherm models to quantitatively describe the interaction between nSiO_2_ and Cd, as well as incomplete characterization of nanoparticle transformations under physiological conditions. It should be emphasized that adsorption kinetic and isotherm models (e.g., Langmuir and Freundlich) represent well-established methodologies in nanomaterial–metal interaction studies. Their application in future investigations would provide essential quantitative parameters for assessing nSiO_2_–Cd binding efficiency and facilitate the predictive accuracy required for scaling up nano-enabled agricultural strategies.

Future research should focus on the following key areas: (1)time-resolved adsorption experiments, surface complexation modeling, and long-term stability assessments of silica nanoparticles within the soil-plant system; (2) long-term toxicity studies to evaluate the persistence and potential ecological impacts of nSiO_2_ in agricultural systems; (3) well-designed field trials to validate the effectiveness of nSiO_2_-assisted phytoremediation under realistic growing conditions; and (4) further investigation into the molecular mechanisms underlying nSiO_2_-mediated Cd sequestration and transport, particularly the roles of specific genes and metabolic pathways identified in this study. From a practical perspective, nSiO_2_-assisted phytoremediation demonstrates promising scalability for real-world application in contaminated farmland. However, several challenges must be addressed before widespread implementation, including the assessment of economic feasibility and comprehensive environmental risks. Despite these challenges, our findings provide valuable insights into the potential of nanotechnology-based approaches for sustainable agriculture and environmental remediation, offering a theoretical foundation for the further development of nanoparticle-assisted phytoremediation strategies.

## Data Availability

The datasets presented in this study can be found in online repositories. The names of the repository/repositories and accession number(s) can be found below: https://www.ncbi.nlm.nih.gov/, PRJNA1272468.
