# Supplementary material for: Silica nanoparticles alleviate cadmium toxicity to Pisum sativum L. seedling growth by remodeling carbon-nitrogen metabolism
Source: Front Plant Sci. 2025 Oct 23;16:1641785. doi: 10.3389/fpls.2025.1641785 (PMC12589072; doi:10.3389/fpls.2025.1641785)
Supplement: Supplementary file 1 [file DataSheet1.docx]

**Supplementary Table** **legends**

Table S1 Statistics of filter quality control results

| Sample | Clean reads | Clean bases | GC Content | %≥Q30 |
| --- | --- | --- | --- | --- |
| L-Control-1 | 21706667 | 6499993216 | 43.08% | 92.60% |
| L-Control-2 | 21556010 | 6153655294 | 43.15% | 92.59% |
| L-Control-3 | 21984352 | 6580795518 | 43.00% | 92.67% |
| L-Cd-1 | 19147902 | 5733904596 | 43.19% | 92.91% |
| L-Cd-2 | 20650997 | 6184351372 | 43.26% | 92.63% |
| L-Cd-3 | 20985076 | 6281020598 | 43.13% | 92.18% |
| L-Cd+nSiO_2_-1 | 21297790 | 6377876192 | 43.18% | 91.85% |
| L-Cd+nSiO_2_-2 | 19260015 | 5766584880 | 42.96% | 92.19% |
| L-Cd+nSiO_2_-3 | 20168765 | 6093316386 | 42.96% | 92.82% |
| L-nSiO_2_-1 | 21897444 | 6556096672 | 43.32% | 94.88% |
| L-nSiO_2_-2 | 20932385 | 6267524802 | 43.29% | 93.04% |
| L-nSiO_2_-3 | 19443800 | 5821425958 | 43.39% | 92.36% |
| R-Control-1 | 22207188 | 6641197810 | 43.04% | 92.84% |
| R-Control-2 | 19508451 | 5835405692 | 42.90% | 91.53% |
| R-Control-3 | 21267672 | 6361833686 | 42.90% | 92.12% |
| R-Cd-1 | 22772054 | 6812627874 | 42.78% | 92.61% |
| R-Cd-2 | 19468530 | 5822292570 | 42.73% | 91.70% |
| R-Cd-3 | 25693913 | 7686154388 | 42.87% | 93.93% |
| R-Cd+nSiO_2_-1 | 19535996 | 5844544580 | 42.66% | 91.37% |
| R-Cd+nSiO_2_-2 | 19859004 | 5939994334 | 42.71% | 92.29% |
| R-Cd+nSiO_2_-3 | 20917372 | 6256983682 | 42.85% | 91.56% |
| R-nSiO_2_-1 | 23308243 | 6973402862 | 42.78% | 92.59% |
| R-nSiO_2_-2 | 20975252 | 6272910134 | 43.67% | 91.91% |
| R-nSiO_2_-3 | 20908453 | 6254565364 | 43.05% | 91.65% |

Note: Samples: Sample analysis number; Clean reads: Total number of pair-end Reads in the Clean Data; Clean bases: Clean Data Total base number; GC content: Clean Data GC Content, that is, the percentage of the two bases, G and C, in the total bases in Clean Data; ≥Q30%: Clean Data Percentage of bases with mass values greater than or equal to 30; L-: leaves; R-: roots.

**Supplementary Figure legends**


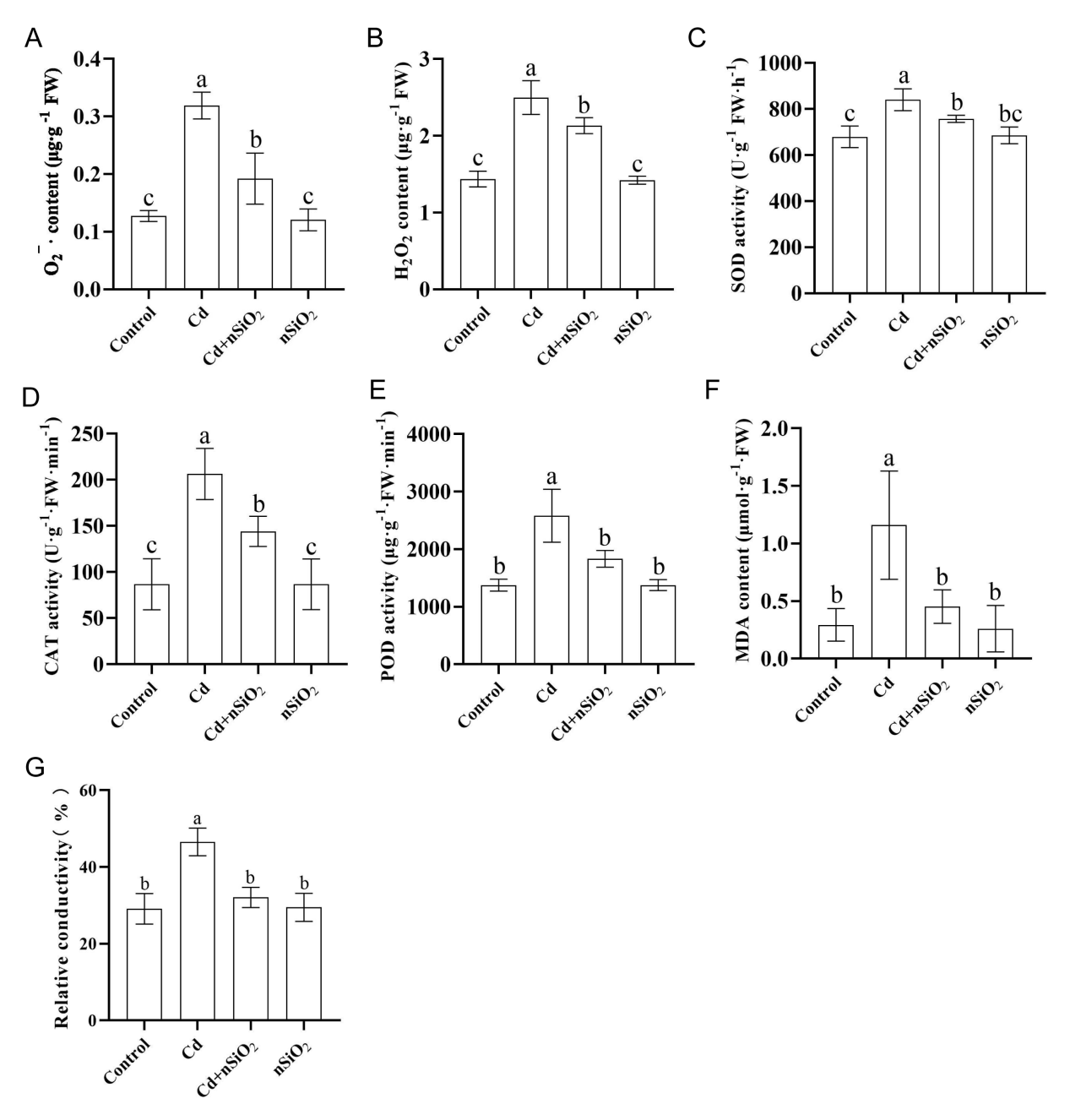


**Figure** **S1** Nano-SiO_2_ exposure alleviated Cd-induced oxidative damage in pea seedling leaves. Two-day-old germinated pea seedlings were transferred to 1/4-strength fresh Hoagland solution with or without 9 μM CdCl_2_, 50 mg/L nSiO_2_ (Control, Cd, Cd+nSiO_2_, nSiO_2_) for 5 days. (**A**) The O_2_¯ levels, (**B**) H_2_O_2_ levels, (**C**) SOD activity, (**D**) CAT activity, (**E**) POD activity, (**F**) MDA levels and (**G**) Relative electrical conductivity were measured. The results are presented as the means ± SDs (*n*=3). Different letters indicate significant differences (*P* < 0.05 according to Tukey’s test).


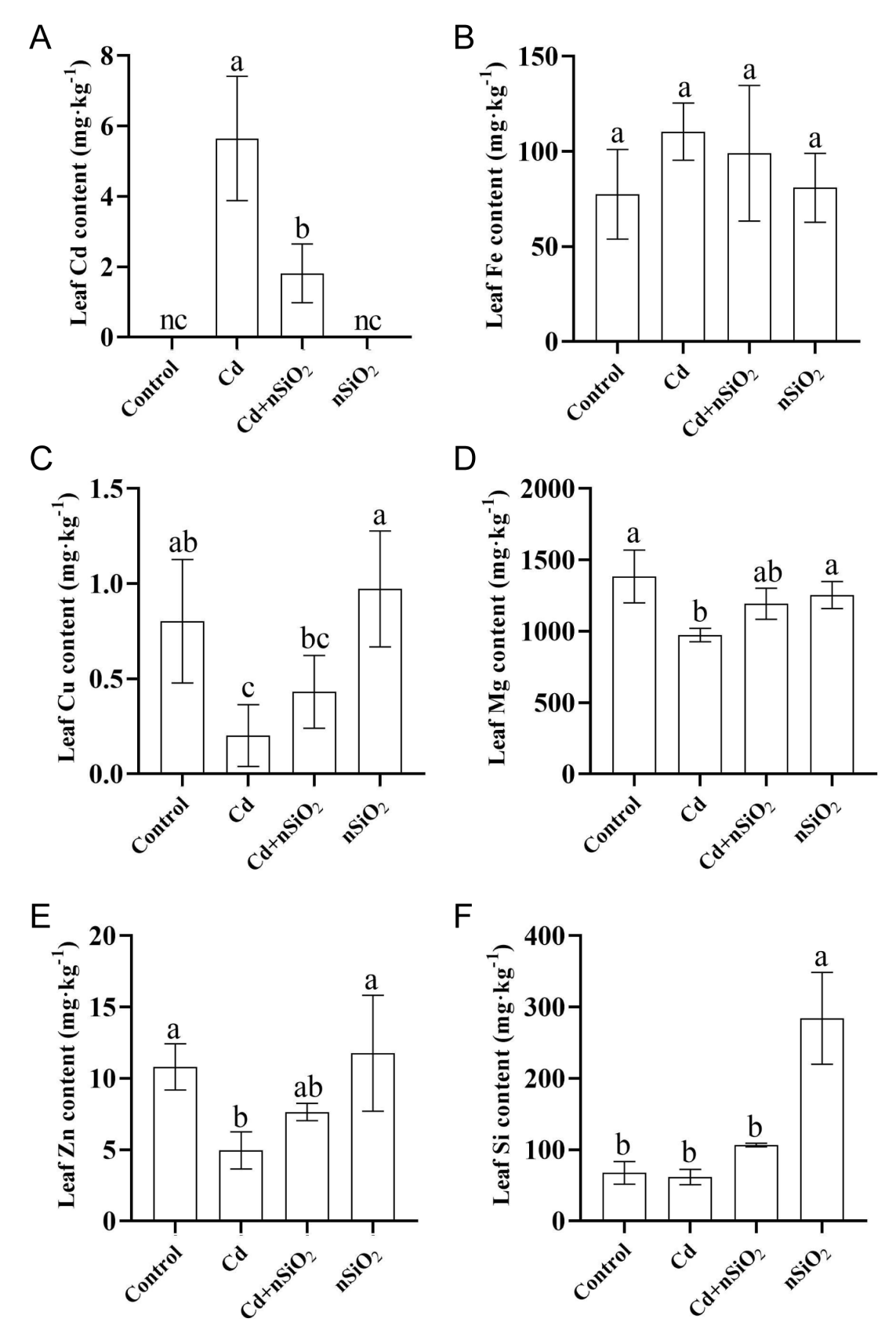


**Figure** **S2** Nano-SiO_2_ exposure reduced Cd accumulation and improved the accumulation of mineral elements in pea leaves under Cd stress. Ten-day-old germinated pea seedlings were transferred to 1/4-strength fresh Hoagland solution with or without 9 μM CdCl_2_, 50 mg/L nSiO_2_ (Control, Cd, Cd+nSiO_2_, nSiO_2_) for 12 days. The concentrations of (**A**) Cd, (**B**) Fe, (**C**) Cu, (**D**) Mg, (**E**) Zn and (**F**) Si were measured. The results are presented as the means ± SDs (*n*=3). Different letters indicate significant differences (*P* < 0.05 according to Tukey’s test).


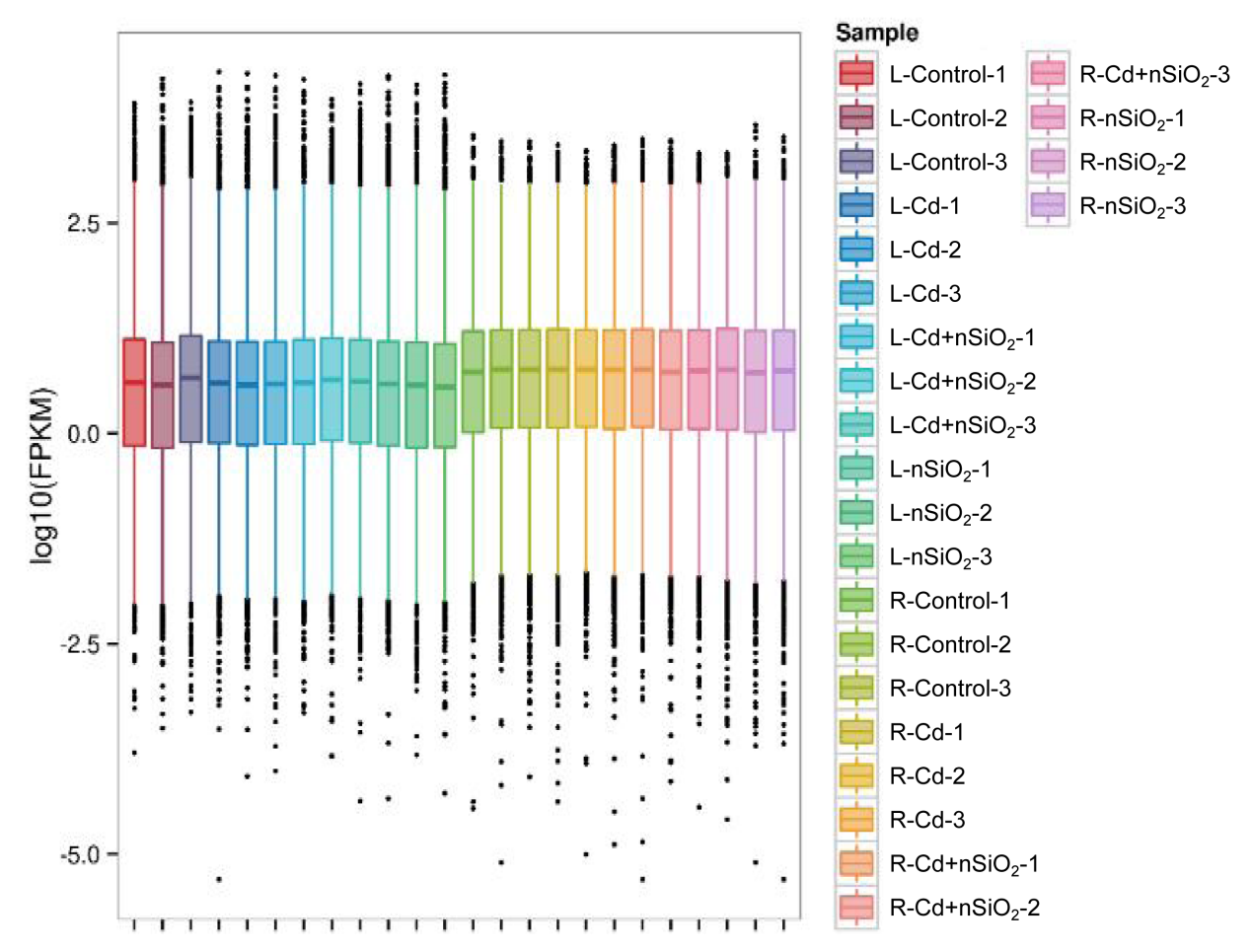


**Figure** **S3** Transcriptome analysis of DEGs in pea seedlings treated with nSiO_2_ or Cd. FPKM boxplots for the different samples. .


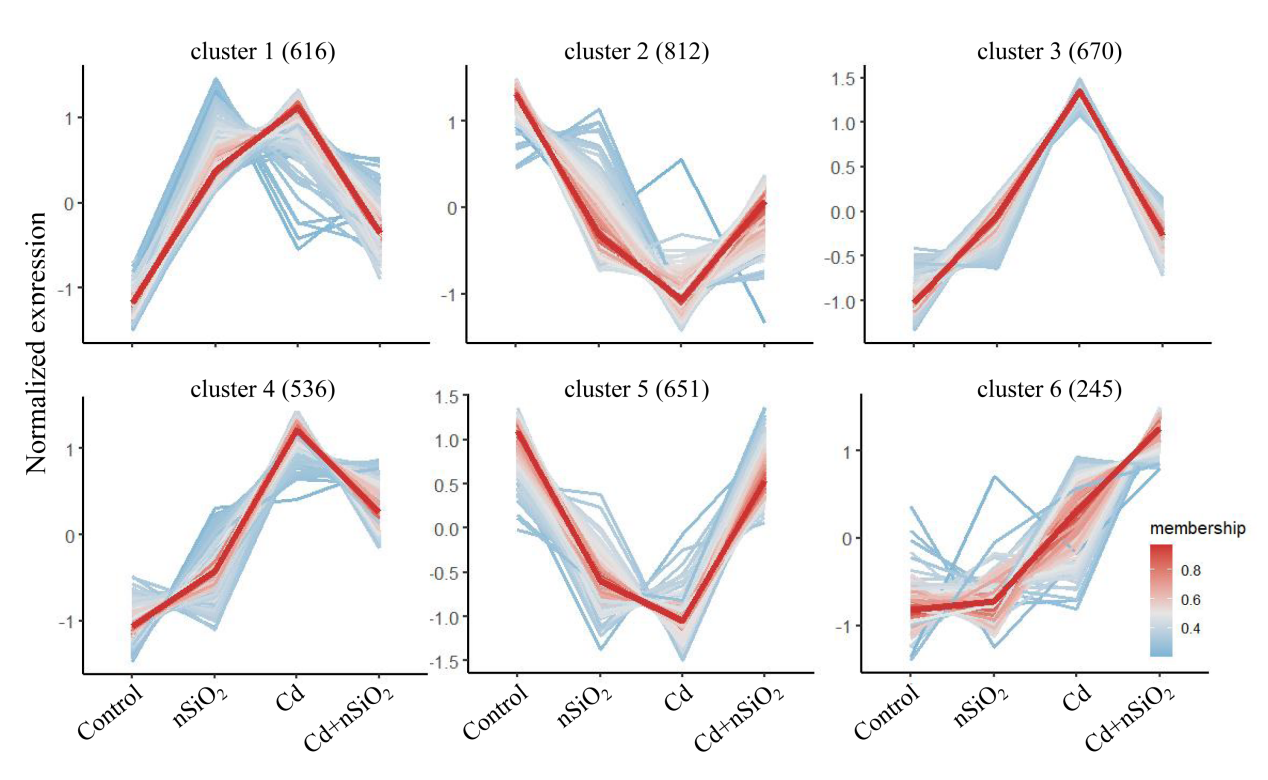


**Figure S4** Perform cluster analysis on DEGs identified in the samples of four groups of pea seedlings under different treatments. Analysis of gene expression trend under different treatments in gene clusters in pea seedlings leaves.


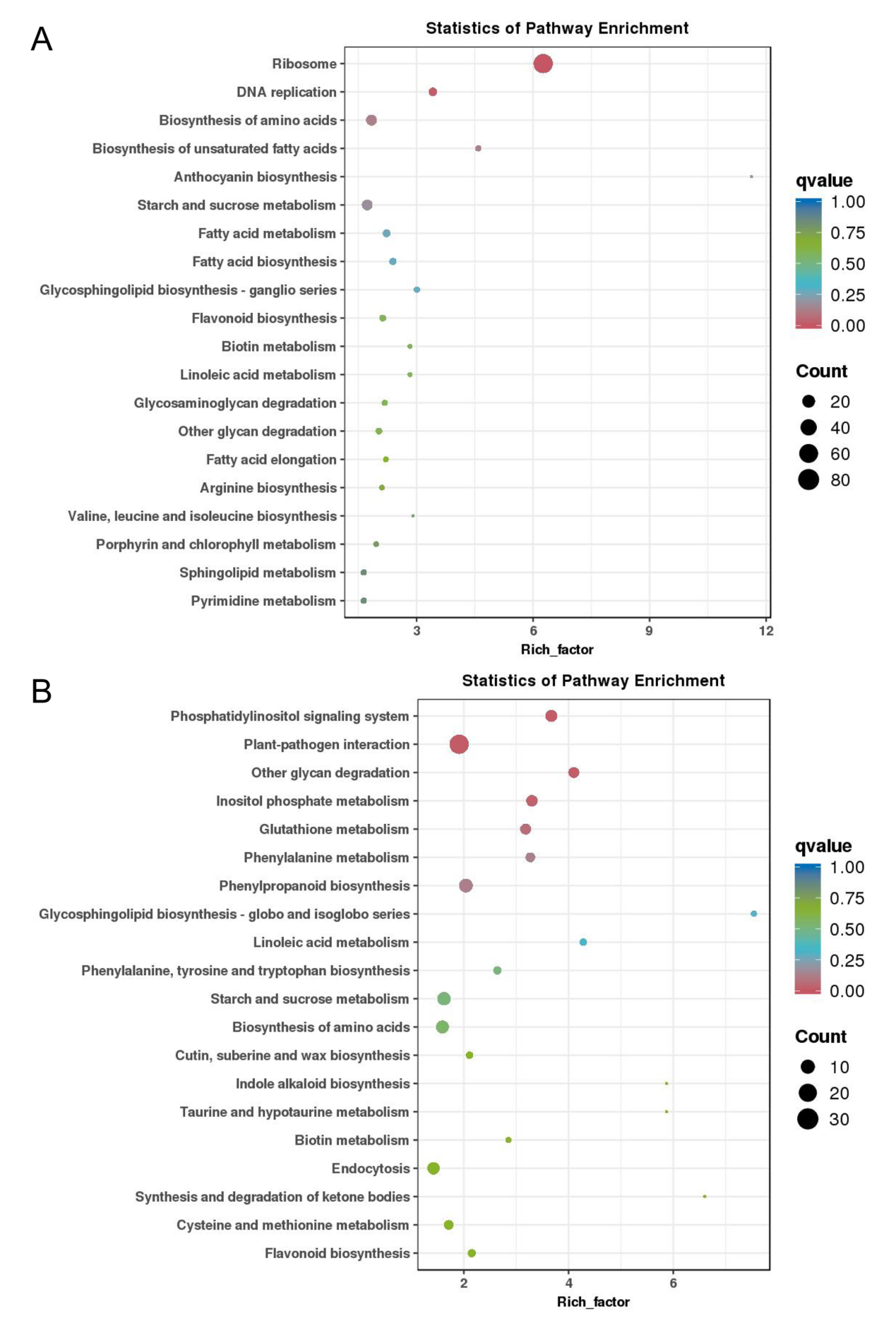


**Figure S5** KEGG enrichment analysis of the differentially expressed genes in pea seedlings. Top 20 enriched KEGG pathways of DEGs from (**A**) cluster 3 and (**B**) cluster 4 in pea seedling leaves.
